# Quantitative Estimation of Leaf Heat Transfer Coefficients by Active Thermography at Varying Boundary Layer Conditions

**DOI:** 10.3389/fpls.2019.01684

**Published:** 2020-01-21

**Authors:** Hendrik Albrecht, Fabio Fiorani, Roland Pieruschka, Mark Müller-Linow, Christoph Jedmowski, Lukas Schreiber, Ulrich Schurr, Uwe Rascher

**Affiliations:** ^1^ Institute of Bio- and Geosciences, IBG-2: Plant Science, Forschungszentrum Jülich, Jülich, Germany; ^2^ Institute of Cellular and Molecular Botany, Department of Ecophysiology, University of Bonn, Bonn, Germany

**Keywords:** active thermography, time constant of cooling, CWSI, leaf transpiration, heat capacity, plant phenotyping

## Abstract

Quantifying heat and mass exchanges processes of plant leaves is crucial for detailed understanding of dynamic plant-environment interactions. The two main components of these processes, convective heat transfer, and transpiration, are inevitably coupled as both processes are restricted by the leaf boundary layer. To measure leaf heat capacity and leaf heat transfer coefficient, we thoroughly tested and applied an active thermography method that uses a transient heat pulse to compute τ, the time constant of leaf cooling after release of the pulse. We validated our approach in the laboratory on intact leaves of spring barley (Hordeum vulgare) and common bean (Phaseolus vulgaris), and measured τ-changes at different boundary layer conditions.By modeling the leaf heat transfer coefficient with dimensionless numbers, we could demonstrate that τ improves our ability to close the energy budget of plant leaves and that modeling of transpiration requires considerations of convection. Applying our approach to thermal images we obtained spatio-temporal maps of τ, providing observations of local differences in thermal responsiveness of leaf surfaces. We propose that active thermography is an informative methodology to measure leaf heat transfer and derive spatial maps of thermal responsiveness of leaves contributing to improve models of leaf heat transfer processes.

## Introduction

Plants continuously interact with their environment by heat and mass exchange and play an important role in the earth’s hydrological and carbon cycle (Foley et al., 2003). The most important physiological process resulting in gas and mass exchange with the atmosphere is photosynthesis accompanied by transpiration. Thus, heat and mass exchange between plants and their environment significantly affects plant productivity, water use, and water use efficiency (Shibuya et al., 2006; Schymanski and Or, 2015). Particularly, water use efficiency is of interest in agriculture for genetic improvement and selection of high-yielding crop varieties for water-limited agriculture (Blum, 2009; Munns et al., 2010; Passioura and Angus, 2010). Consequently, studying plants heat and mass exchange is key to understand the dynamics of plant-environment-interactions.

An important component of plant heat exchange is the convective heat transfer, the exchange of heat between the leaf surface and the surrounding atmospheric conditions. Heat penetrates the boundary layer of a leaf that is characterized by gradients of temperature, gas concentrations, and air velocities determining the boundary layer conductance (Raschke, 1960; Schuepp, 1993; Schreuder et al., 2001; Vogel, 2009). Because transpiration, i.e., heat loss via water vapor, affects leaf temperature (T_L_) (Gates, 1968), and T_L_ also affects convective heat transfer (Dixon and Grace, 1983), convection and transpiration are inevitably coupled to each other. Additionally, transpiration is constrained by boundary layer conductance. Wind also affects transpiration rates by removing the water vapor within the boundary layer leading to a higher leaf-to-air water vapor pressure deficit and ultimately may induce stomatal closure (Grace, 1974; Dixon and Grace, 1983; Bunce, 1985).

Because transpiration, i.e., heat loss via water vapor, affects leaf temperature (T_L_) (Gates, 1968), which also affects convective heat transfer (Dixon and Grace, 1983), convection and transpiration are closely coupled. Additionally, stomatal response to environmental conditions such as humidity or CO2 concentration further affects these exchange rates (Grace, 1974; Dixon and Grace, 1983; Bunce, 1985). The boundary layer conductance cannot be measured directly and it is often approximated by heat transfer coefficients that relate heat flux densities per unit leaf area to the difference between T_L_ and ambient air temperature (T_L_-T_a_) (Raschke, 1960; Schuepp, 1993). Heat transfer coefficients depend largely on air speed (forced convection) or temperature differences (free convection), which can be further approximated with dimensionless numbers (Defraeye et al., 2013). Usually both free and forced convection occur under a wide range of conditions determining the heat exchange processes to a different extent depending on the prevailing conditions (Dixon and Grace, 1983; Monteith and Unsworth, 2008; Nobel, 2009).

Heat and mass exchange processes are also affected by incoming radiation and thermal conductivity. While radiation is an important factor influencing photosynthesis and transpiration (Roelfsema and Hedrich, 2005; Pieruschka et al., 2010; Mott and Peak, 2011), variation of thermal conductivity is often neglected in quantitative analyses because thermal conductivity of plant leaves is generally low (Jayalakshmy and Philip, 2010). The low thermal conductivity of plant leaves is a result of a relatively high content of water resulting in a high specific heat capacity. Water is the main component of leaves and considerably affects the leaf heat capacity. Therefore, the leaf water content per unit area (LWC), the leaf succulence, affects the dynamics of T_L_ response to heat absorption, which, in turn, affects the dynamics of convection, and transpiration (Gates, 1968; Dixon and Grace, 1983; Bailey and Meneses, 1993). The thermal responsiveness of a leaf, which describes to what extent and how fast a leaf heats up or cools down, depends on leaf heat capacity and leaf heat transfer coefficient (hleaf), which both can be estimated by the time constant (τ) of T_L_ dynamics. The time constant is characterized by the dynamic response of T_L_ to different environmental factors with τ as the product of leaf heat capacity and the inverse of the heat transfer coefficient (Jones, 1992; Monteith and Unsworth, 2008; Nobel, 2009).

The use of τ as a measure of the thermal responsiveness of leaves and for hleaf modeling was proposed in early studies in the 60’s using the so-called “cooling curve technique” (Linacre, 1972; Pearman et al., 1972). According to this method, the temperature of leaves or artificial leaf models, is transiently increased by a short (seconds) heat pulse and the following cooling curve is recorded. This temperature cooling kinetic provides τ, which is the slope of the exponential decay curve. For example, Linacre, 1972 used wet blotting paper to estimate the heat transfer of transpiring leaves and Pearman et al. (1972) used copper discs to estimate leaf heat transfer in a canopy under field conditions. However, artificial leaves have a different thermal conductivity compared to leaf tissue. For example, the thermal conductivity of copper (400 W m^-1^ K^-1^) is substantially greater than that of water (0.6 W m^-1^ K^-1^) or leaves (0.2 to 0.5 W m^-1^ K^-1^) (Jayalakshmy and Philip, 2010). Large thermal conductivity also allows lateral heat conduction, which is very low in leaf tissue. Finally, the heat capacity of different materials used as artificial references is also not comparable to that of leaves leading to incorrect estimation of τ-values for real leaves.

Studies using the cooling curve technique on intact leaves were mainly performed using thermocouples or radiometers for T_L_ measurements (Parlange and Waggoner, 1971; Saldin and Barthakur, 1971; Pearman et al., 1972). Assessment of TL by using thermocouples is not fully non-invasive, because thermocouples are attached to the leaf surface (Kumar and Barthakur, 1971) and therefore may affect the leaf heat transfer. Radiometers may be preferable, because these sensors are not attached to the leaf and therefore do not disturb the leaf heat transfer. However, radiometers do not provide spatial information of T_L_. Recently, non-invasive thermal imaging has become a powerful alternative to point measurements. To our knowledge, there is only one study in which τ was derived by using thermography on plant leaves. Similar to the cooling curve technique Kümmerlen et al. (1999) used the “active thermography” approach and measured intact leaves enclosed in a gas exchange cuvette. These authors were able to derive LWC from τ-measurements and hleaf from gas exchange measurements in Ricinus comminus plants.

To model leaf heat transfer processes in leaves the implementation of τ is very relevant (Leigh et al., 2012). We have revisited this type of measurements using an active thermography protocol applying short infra-red radiation pulses and tested the thermal responsiveness of contrasting leaf types (i.e., leaf structure, vascular tissue) of spring barley (Hordeum vulgare) and common bean (Phaseolus vulagris). We modeled hleaf response to wind and varying irradiance by using τ and dimensionless numbers and hypothesized that τ decreases in response to wind with an increase in h_leaf_. Additionally, based on pixel-wise computation of τ from time series of thermal images, we provide spatial maps of leaf thermal responsiveness. Those spatial maps allow to separate areas in which thermal responsiveness is mainly driven by leaf heat capacity from areas where thermal responsiveness is mainly driven by heat transfer processes. We suggest that the active thermography approach can be a powerful tool for modeling leaf heat transfer under well-defined environmental conditions in the laboratory.

## Material and Methods

### Theoretical Background and Model Description

According to the commonly used description of the steady state leaf energy balance model, all absorbed heat that originates from absorption of solar and thermal radiation (Φ_in_), is dissipated by heat flux densities (W m^-2^), which are namely the long-wave radiative heat flux density (LW), the convective heat flux density (H), and the evapotranspiration (λE) (Linacre, 1972; Jones, 1992; Monteith and Unsworth, 2008; Nobel, 2009) for abbreviations see **Table 1**.

**Table 1:** Abbreviation list.

Abbreviation	Description	Unit
C A^-1^leaf	leaf heat capacity per unit area	J m-^2^ K^-1^
c_p_	specific heat capacity at constant pressure	J kg-^1^ K^-1^
d	characteristic leaf geometry	m
gH	boundary layer conductance to convective heat	m s-^1^
g_LW_	boundary layer conductance to radiative heat	m s-^1^
Gr	Grashof number	
g_s_	stomatal conductance	m s-^1^ or mol m-^2^ s^-1^
H	convective heat flux density	W m^-2^
h_H_	convective heat transfer coefficient	W m^-2^ K^-1^
h_leaf_	leaf heat transfer coefficient	W m^-2^ K^-1^
h_LW_	long-waver radiative heat transfer coefficient	W m^-2^ K^-1^
h_λE_	evapotranspirative heat transfer coefficient	W m^-2^ K^-1^
k	thermal conductivity of air	W m^-1^ K^-1^
LW	long-wave radiative heat flux density	W m^-2^
LWC	leaf succulence/leaf water content per unit area	mg cm^-2^
Nu	Nusselt number	
r_aW_	boundary layer resistance to water vapor	s m^-1^
Re	Reynolds number	
r_H_	boundary layer resistance to convective heat	s m^-1^
r_s_	stomatal resistance	s m^-1^ or m² s mol^-1^
r_W_	leaf resistance to water vapor (raW + rs)	s m^-1^
s	slope relating saturation vapor pressure to temperature	Pa K^-1^
t_0.5_	time which is required to reach 50% of the initial value in an exponential decay	s
T_a_	ambient air temperature	°C or K
T_L_	steady state leaf temperature	°C or K
T’_L_	non-steady state leaf temperature	°C or K
T_L_-T_a_	difference leaf temperature to ambient air temperature	°C or K
u_0.5_	wind speed at which τ has decreased to 50% of its initial value	m s^-1^
γ	psychrometer constant	Pa K^-1^
ϵ	emissivity	
λE	evapotranspiration	W m^-2^
ρ_a_	density of air at constant pressure	kg m^-3^
τ	time constant	s
Φ_in_	incoming radiation (solar radiation + thermal radiation)	W m^-2^

(1) $$0={\text{ Φ}}_{\text{in}}-\text{LW}-\text{H}-\text{λE}$$

If a leaf is not in an equilibrium, for instance following a short heat pulse, T_L_ transiently changes and afterwards approaches its former steady state value. To what extent a leaf heats up and how rapidly the heat is released, depends to a large extent on the leaf heat capacity per unit area ($C\;A_{leaf}^{-1}$), which is the energy required per leaf area to heat up the leaf by one degree. The difference between the leaf energy balance in equilibrium and in non-equilibrium is described as follows [for a derivation of the non-equilibrium leaf energy balance, see for example Appendix 9 in Jones (1992)]:

(2) $${\frac{\text{C}}{\text{A}}}_{\text{leaf}}\frac{\Delta {\text{T}}_{\text{L}}}{\Delta \text{t}}={\text{ρ}}_{\text{a}}{\text{c}}_{\text{p}}\left({\text{T}}_{\text{L}}^{\prime }-{\text{T}}_{\text{L}}\right)\left[{\text{g}}_{\text{LW}}+{\text{g}}_{\text{H}}+\left(\frac{\text{s}}{{\text{γr}}_{\text{W}}}\right)\right]$$

With ΔT_L_ Δt^-1^ as the T_L_ change over time, ρ_a_ is the density of air, c_p_ the specific heat capacity of air, T’_L_ the leaf temperature in non-steady state, T_L_ the steady-state temperature, g_LW_ the conductance to long-wave radiative heat, g_H_ the conductance to convective heat, s the slope relating saturation vapor pressure to air temperature (Penman, 1948), γ the psychrometer constant in Pa K^-1^ which changes with temperature (e.g., Table A.3 in Monteith and Unsworth, 2008), and rW the resistance to water vapor, which is the sum of the boundary layer resistance to water vapor (r_aW_) and stomatal resistance (r_s_). The formulation for r_aW_ accounts for amphistomatous leaves, such as barley and bean. For hypostomatous species this formulation differs slightly (see, e.g., Jones, 1992).

Because, the product of ρ_a_, c_p_ and g is known as the heat transfer coefficient (h), Eq. 2 can be written as:

(3) $$\frac{C}{A_{leaf}}\frac{\Delta T_{L}}{\Delta t}=\left(T_{L}^{\prime }-T_{L}\right)h_{leaf}$$

Where h_leaf_ is the total leaf heat transfer coefficient and is the sum of the heat transfer coefficient for long-wave radiative heat (h_LW_), the heat transfer coefficient for convective heat (h_H_), and the heat transfer coefficient for evapotranspirative heat (h_λE_).

(4a) $$h_{leaf}=h_{LW}+\;h_{H}+\;h_{\lambda E}$$

(4b) $$h_{LW}=\;\rho_{a}c_{p}g_{LW}$$

(4c) $$h_{H}=\;\rho_{a}c_{p}g_{H}$$

(4d) $$h_{\lambda E}=\;\rho_{a}c_{p}\left(\frac{s}{\gamma r_{W}}\right)$$

The gLW is given by $4\epsilon \sigma T_{a}^{3}\;\rho_{a}^{-1}cp^{-1}$ with *ϵ* being the emissivity and σ the Stefan-Boltzmann constant. As stated above, r_W_ is the sum of r_aW_ and r_s_, where r_aW_ is assumed to be approximately r_H_, the resistance to convective heat, as the reciprocal of g_H_ (Monteith and Unsworth, 2008).

To solve Eq. 3, a first-order differential equation is used, which is in a form of Newtons law of cooling (e.g., see Chapter 15 and Eq. 15.10 in Monteith and Unsworth, 2008):

(5) $$dT_{L}\left(t\right)=\frac{1}{{\frac{C}{A}}_{leaf}h_{leaf}}\left(T_{L}^{\prime }-T_{L}\right)$$

Note, that the application of Newton’s law of cooling assumes constant ambient conditions.

In a further step, Eq. 5 can be solved with the following exponential function (**Figure S1**):

(6) $${\text{dT}}_{\text{L}}^{*}={\text{T}}_{\text{L}}-\left({\text{T}}_{\text{L}}-{\text{T}}_{\text{L}}^{\prime }\right){\text{e}}^{-\frac{\text{t}}{\tau }}$$

Where $T_{L}^{*}$ is any T_L_ during leaf cooling and τ the time constant, which is according Eq. 5 the product of leaf heat capacity per unit leaf area and the inverse of the leaf heat transfer coefficient:

(7) $$\tau =\;{\frac{\text{C}}{\text{A}}}_{\text{leaf}}\frac{1}{h_{leaf}}={\frac{\text{C}}{\text{A}}}_{\text{leaf}}\frac{1}{{\text{ρ}}_{\text{a}}{\text{c}}_{\text{p}}\left({\text{g}}_{\text{LW}}+{\text{g}}_{\text{H}}+\left(\frac{\text{s}}{{\text{γr}}_{\text{W}}}\right)\right)}$$

Using dimensionless numbers, gH can be calculated with the following equation (e.g., Dixon and Grace, 1983; Bailey and Meneses, 1993):

(6) $${\text{g}}_{\text{H}}=\frac{\text{Nu k}}{{\text{ρ}}_{\text{a}}{\text{c}}_{\text{p}}\text{d}}$$

Nu is the Nusselt number, k the thermal conductivity of air, and d refers to the mean leaf diameter in m.

Nu depends on the prevailing conditions, in particular whether free or forced convection is dominant. Under free convection, where no wind occurs and heat transfer is mainly due to heat upwelling from the leaf surface, Nu depends on a further dimensionless number, the Grashof number (Gr):

(7) $$\text{Nu}={\text{aGr}}^{\text{b}}$$

The numerical constants a and b describe the geometry of a leaf (Schuepp, 1993).

Under conditions where wind occurs, a further dimensionless number has to be considered, the Reynold’s number (Re), which describes forced convection. At low wind conditions, mixed convection is most likely (Schuepp, 1993) and Nu has to be calculated with Gr and Re.

(8) $$\text{Nu}=\text{a}{\left(\text{Gr}+1.4{\text{Re}}^{2}\right)}^{\text{b}}$$

We determined a and b experimentally for both barley and bean leaves with respect to the prevailing wind conditions. Detailed descriptions of the model we used and the respective values are given in Supplementary material (Eq. S1 to Eq. S8, **Table S1**, and **Figures S1** to **S4**).

### Plant Material

All plants were grown in the greenhouse facilities at the IBG-2, Forschungszentrum Jülich in spring 2015. We grew spring barley (*Hordeum vulgare* var. Victoriana) and common bean (*Phaseolus vulgaris* var. Shiny). Barley plants were germinated in 12 x 12 x 15 cm pots and bean plants in 15 x 15 x 18 cm pots. Pots were filled with a potting substrate, enriched with 1 g L^-1^ NPK fertilizer and with 2 g L^-1^ of a long-time acting fertilizer (Einheitserde Typ ED73). Plants were placed on moist, water-retentive cloths.

Plants grew in a day-night cycle of 16 h day and 8 h night with air humidity around 55% ± 13%. Mean T_a_ was 20.8°C ± 2.6°C, and the highest measured T_a_ during this period was 30.7°C, whereas the minimum T_a_ was 16.8°C. On sunny days, light intensities in the greenhouse at midday reached on maximum about 1,300 µmol m^-2^s^-1^, while the minimum illumination intensity was about 85 µmol m^-2^ s^-1^, including artificial light, in the late afternoon. For the measurements, bean plants were about 2 weeks old and barley plants were about 6 weeks old.

For all measurements, plants were moved from the greenhouse into the laboratory, where they were dark-adapted over night for about 14 h prior to the measurements.

### Thermal Imaging

For all measurements we used a VarioCAM ® hr head (InfraTec, Germany). This camera is equipped with a microbolometer focal plane array that captures and integrates thermal infrared radiation within the spectral range between 7.5 µm and 14 µm. The field of view (FOV) is 30° x 23°, with a geometric resolution of 640 by 480 pixels, a measuring accuracy of ± 1 K, with a thermal sensitivity <30 mK. Images were recorded with the IRBIS ® 3 software (InfraTec, Germany) that allows real-time tracking and correction of temperatures by setting parameters, such as ϵ and background radiation. Background radiation was measured by a sheet of crumpled aluminum foil and emissivity (ϵ) was set to 0.95 (Nobel, 2009). Even though the radiator beam was mainly targeting the leaf itself during the measurements, the background temperature showed slight variations in the range of 0.3–0.4 K, as measured by aluminum foil placed in the center of the beam. According to the Stefan-Boltzmann Law this corresponds to a variable incoming background energy of approx. 2 W m^2^ and we assume that these small variations had only a minor effect on the time constant of leaf cooling. The IRBIS software allows the export of single pixel and integrated pixel area data, as well as pixel data as a text file.

To adapt T_a_, measured by thermocouples (Type K, Newport Omega, Germany) to temperatures measured by thermography, we developed a correction routine for T_a_. For this purpose, we built a “radiation-trap” which absorbs and emits all incoming heat, providing a black-body-like reference. We used a 2x2x15 cm box made of black cardboard, which was thermally insulated by means of a 0.5 cm thick Styrofoam layer to minimize temperature fluctuations. The inner side of the box was covered with aluminum foil, which was crumpled and painted with black emissivity paint (ϵ = 0.95) (TETENAL Europe GmbH, Germany), so that the overall emissivity of the inner box was 1. The upper side of the box, always facing the camera, had a 1-cm-diameter hole. Within the box a thermocouple was placed, which is identically constructed to the one that measures T_a_ outside the box. To adapt the measured T_a_ to temperatures obtained by thermography, we calculated the difference between the thermocouple within the box and the temperature of the hole measured with the thermal camera. In preliminary experiments, we compared T_L_-T_a_ obtained by thermography with T_L_-T_a_ obtained by thermocouples and found that we could use the box as a correction factor for T_a_.

To actively and transiently warm up leaves with short heat pulses, we used two commercial near-infrared (NIR) heating units (Heizmeister 1000 IP65, Infralogic, Germany) equipped with a “long life light-tube (Helen Goldröhre”, Infralogic, Germany), which emits radiation with a maximum power of 1,000 W m_-2_ in the range between 750 and 2,000 nm. This spectral range is suitable for actively heating leaves because significant water absorption bands located at 1,450 and 1,950 nm within this spectral range (Gausman and Allen, 1973; Asner, 1998; Seelig et al., 2008; Seelig et al., 2009). The heating units were connected to unit (Eurolite ® ESX-4 DMX, Eurolite, Germany) that was software controlled (DMX-Configurator, DMX4ALL GmbH, Germany) allowing the setting of intensity, duration, and interval of the NIR heat pulses. We applied a 1.15 s heat pulse to the leaves at approximately half of the maximum power.

### Experimental Set-Up

All instruments were mounted on a metal profile construction (**Figures S6** and **S7**). The camera head was mounted at a height of 1 m at a 90° angle to the ground. The two NIR heaters were placed at the camera’s height with a distance of about 30 cm to the camera and facing to the ground at an angle between 60° and 70°. Additionally, two white LED panels (SL 3500-W-G, Photon System Instruments, Czech Republic) were installed at the camera’s height at an angle of about 45° to the ground. A small ventilator, capable to produce low wind-speeds between 0.2 and 1.6 m s^-1^, was installed on a vertical metal bar, in order to produce laminar wind streams from the leaf tip to the leaf base. Wind-speeds were measured by a thermo-anemometer with hotwire (VT 110, KIMO Instruments, France). Leaves were fixed by lab-stand clamps horizontally to the ground, i.e. perpendicular to the camera. T_a_ was measured with a thermocouple that was attached to the lab-stand and protected against direct irradiation with aluminum foil. T_a_ and the temperature within the radiation trap were recorded every second with a data logger (HH506RA, Newport Omega, Germany).

### Measurements

#### Leaf Water Content

For the measurements with barley, leaves of different developmental stages were randomly chosen. Measurements with bean, were performed with the primary leaf pair and plants were not older than 2 weeks, because leaves at different developmental stages showed different leaf shapes, which in theory impacts the leaf heat transfer. Intact leaves were dark-adapted and measured at three different wind-speeds, 0.0 m s^-1^, 0.5 m s^-1^, and 1.0 m s^-1^. For each wind-speed step, 15 barley leaves and 8 bean leaves each of different plants were measured. After T_L_ cooling curves were measured and leaves had cooled to T_L_ prior to the heat pulse, stomatal conductance (g_s_) was measured with a gas exchange device (Licor-6400, LICOR, Nebraska, USA. Leaves were harvested and analyzed for leaf area and fresh weight afterwards. For dry weight determination, harvested leaves were dried in an oven at 80°C for 48 h until a constant weight was reached. We calculated LWC, the absolute leaf water content per unit area, as the difference between fresh weight and dry weight per unit leaf area.

### Wind Treatments

To induce changes in the boundary layer thickness and conductance, single leaves were exposed to increasing wind speeds, which were produced by the small ventilator integrated in the set-up. Reference measurements for gH modeling were performed with dark-adapted leaves at eight wind-speed steps, 0.0, 0.2, 0.4, 0.6, 0.8, 1.0, 1.2, and 1.4 m s^-1^ on a separate set of plants. For the actual experiment, a second set of plants was used. Dark-adapted leaves were exposed to increasing wind at speeds of 0.0, 0.4, 0.8, and 1.2 m s^-1^ and measured at each step. Afterwards, the leaves were light-adapted to a light intensity (photosynthetically active photon flux density) of about 1000 µmol m-2 s^-1^, using white LED panels, until T_L_ and stomatal conductance (g_s_) reached steady state values. Usually this was the case after 30 to 40 min after light exposure. Leaves were measured again at the four wind speeds indicated above. Again, g_s_ was measured using the Licor-6400 after each leaf cooling curve. Typically, g_s_ stabilized and reached stable values within 1 min.

### Data Processing and Analyses

#### τ-Measurements

We evaluated measured cooling curves using two procedures. In a first procedure, we used the mean T_L_ values, which were obtained by defining the whole leaf as an area of interest (IRBIS® 3 software, InfraTec, Germany. The software automatically integrates all temperature pixels and provides the mean T_L_ value. Measured cooling curves were then fitted with Eq. 6 to obtain τ from the fitting (Origin 8.5, OriginLab, USA).

In a second procedure, we mapped τ spatially by calculating τ for each single pixel in the image. For this purpose, we developed an automated analysis routine for the MATLAB Environment. A typical data set consists of n images, containing a data matrix with the temperatures *T^t^* at the measured time *t*. The fitting function $T_{L}{\left(t\right)}_{ij}=T_{L}{\left(t_{\infty }\right)}_{ij}-dT_{L_{ij}}e^{-\left(t\;\tau^{-1}\right)}$ was computed for each pixel at the position ij and is in the form of Eg. 3. The optimization of the curve fitting was done by minimizing the sum of squared residuals using the downhill simplex approach (Nelder and Mead, 1965).

A graphical user interface (GUI) supports the processing of a single image series, or of several image series. The required input data are an Excel-file containing the time points of each recorded image and the corresponding images as a text file (ASCII), which were exported from the IRBIS® 3 software before. For the data import of several image series, a list (Excel-file) referring to the respective file path is required. Images and time data, which are located in the respective file-path, are then automatically loaded. Additionally, the GUI provides functions for postprocessing of τ-matrices. Minimum- and maximum-thresholds for τ-values can be used. These were generally set between 0 s and 250 s. Additionally, the r-value of the exponential regression can be used as a further filter-parameter, which we have set to r = 0.9487, which corresponds to a r²-value of 0.9. Resulting filtered as well as non-filtered τ-matrices are provided as Excel-tables for further manual post-processing. The thresholds used in the postprocessing procedure may result in empty pixels on the imaged leaf, which were filled by using the median-value of the surrounding pixels.

### Statistical Analyses

Statistical analyses were performed using SigmaPlot (Systat Software, Inc., USA) and included analyses of variance (ANOVA), Pearson correlation analyses for linear relationships, and Spearman correlation analyses for nonlinear relationships. Before each ANOVA, the data were tested for normal-distribution. In cases where no normal-distribution was present, an ANOVA on the ranks was performed. In each case, the Tukey post-hoc test was used for pairwise comparisons.

## Results

### Relationship Between LWC and τ of Dark-Adapted Leaves

**Figure 1:**
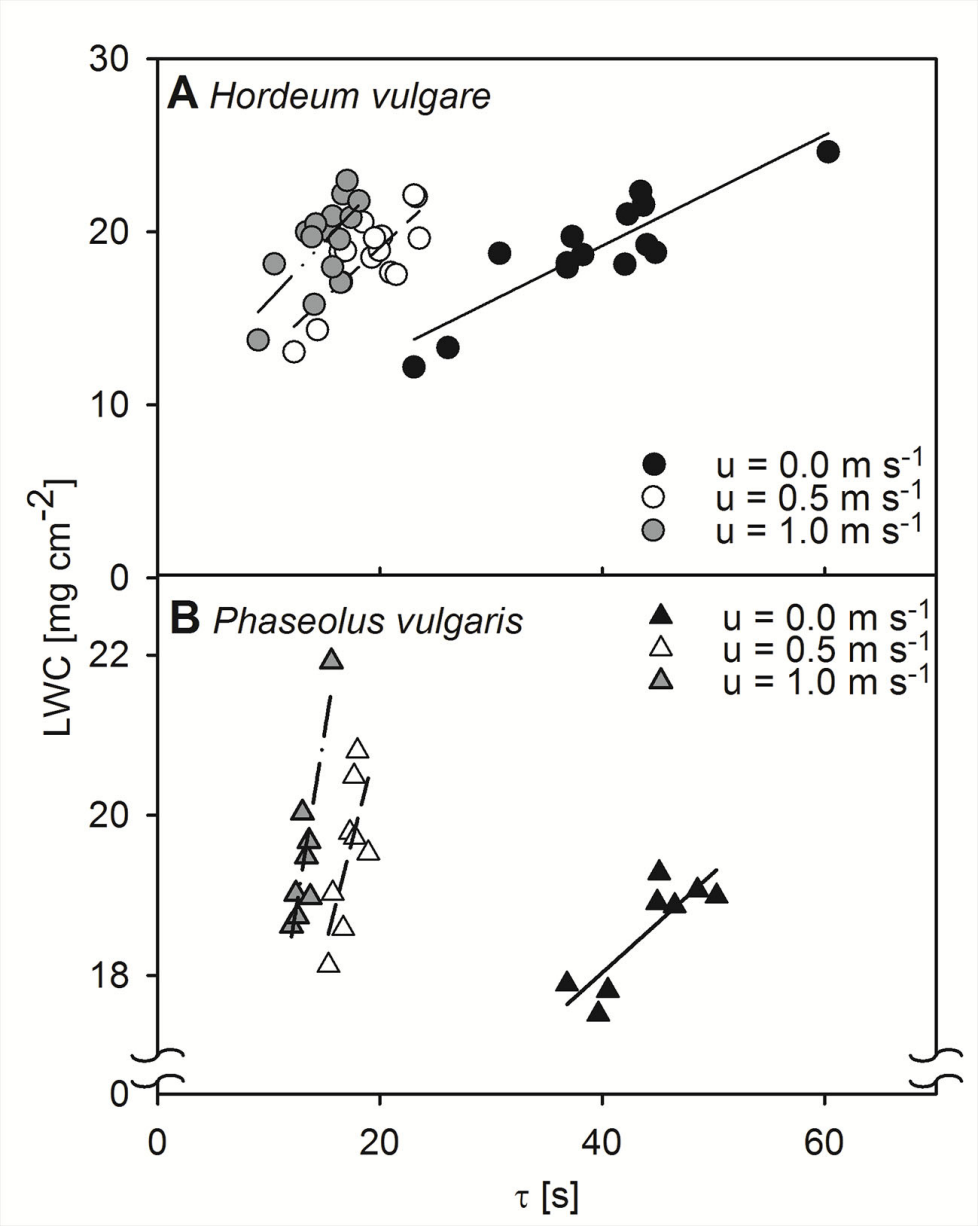
Relationship between leaf water content per unit area (LWC) and time constant (τ) of dark-adapted leaves. Relationships were assessed at different wind speeds of 0.0 m s^-1^ (closed symbols), 0.5 m s^-1^ (open symbols), and 1.0 m s^-1^ (grey symbols). **(A)** Measurement of single leaves of spring barley (Hordeum vulgare). Linear regression for measurements at a wind speed of 0.0 m s^-1^ (solid line): y = 0.32x + 6.40, r² = 0.79, p < 0.001, for measurements at wind speeds of 0.5 m s^-1^ (dashed line): y = 0.59x + 7.29, r² = 0.63, p < 0.001, and for measurements at wind speeds of 1.0 m s^-1^ (dash-dot-dotted line): y = 0.68x + 9.18, r² = 0.48, p < 0.05. **(B)** Measurements of single leaves of common bean (Phaseolus vulgaris). Linear regression for measurements at a wind speed of 0.0 m s^-1^ (solid line): y = 0.12x + 13.04, r² = 0.72, p < 0.01, for measurements at wind speeds of 0.5 m s^-1^ (dashed line): y = 0.54x + 10.17, r² = 0.51, p < 0.05, and for measurements at wind speeds of 1.0 m s^-1^ (dash-dot-dotted line): y = 0.86x + 8.14, r² = 0.79, p < 0.01. Each point represents an individual leaf that was measured by the active thermography approach and afterwards destructively analyzed for LWC.

We tested the relationship between τ, obtained by active thermography, and LWC by measuring dark-adapted leaves of both, spring barley and common bean. These relationships were assessed at three different wind-speeds, 0.0 m s^-1^, 0.5 m s^-1^, and 1.0 m s^-1^ (**Figure 1**). In all cases, correlations between τ and LWC were significant for both barley and bean, at all wind speed steps (p < 0.05). Generally, the linear regression to quantify the relationships revealed slopes that increased with increasing wind speed.

### Wind- and Light-Induced Changes in Leaf Heat Transfer Parameters

**Figure 2:**
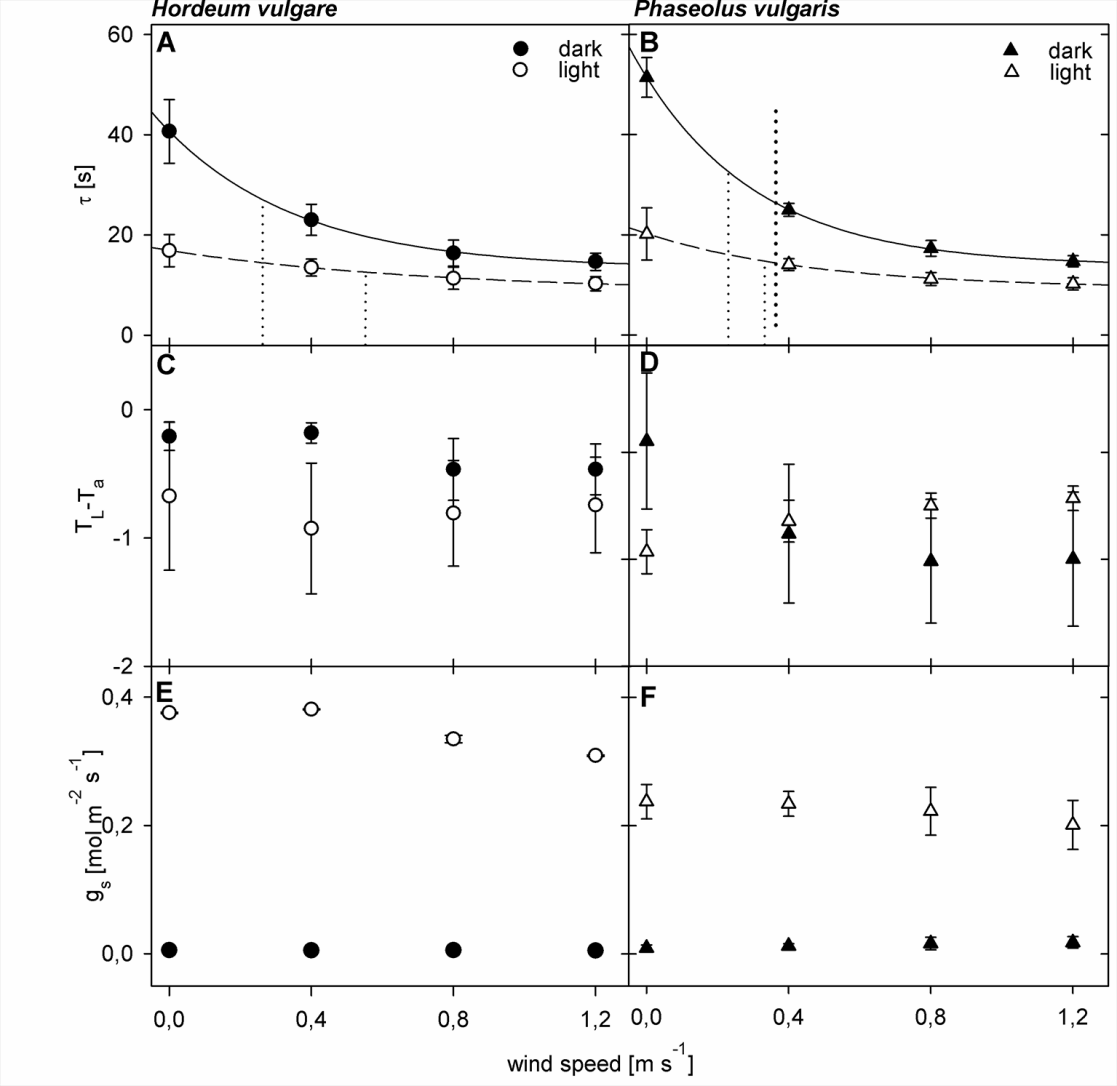
Wind- and light-induced changes in leaf heat transfer parameters of spring barley (Hordeum vulgare) and common bean (Phaseolus vulgaris). Measurements of dark-adapted leaves are indicated by closed symbols, and measurements of light-adapted leaves are indicated by open symbols. **(A**, **B)** Wind- and light induced changes in time constant (τ). Dotted lines represent the wind speed at which τ has decreased to 50% of its initial value (u_0.5_) **(C**, **D)** Wind- and light-induced changes in difference between leaf temperature and ambient air temperature (T_L_-T_a_). **(E**, **F)** Wind- and light-induced changes in stomatal conductance (g_s_). Error bars indicate standard deviation. For barley plants n = 9 individual leaves, for bean n = 10 individual leaves.

To quantify the effect of a changing boundary layer on τ, we compared wind curves of dark-adapted leaves to wind-curves of light-adapted leaves (**Figure 2**). For both barley and bean we observed significant changes (p < 0.05) in τ for dark- and light-adapted leaves in response to increasing wind speed (**Figures 2A, B**). We characterized the τ-response with an exponential regression and the derived wind speed at which τ has decreased to 50% of its initial value (u_0.5_). For dark-adapted leaves we obtained u_0.5_-values of 0.26 m s^-1^ and 0.23 m s^-1^ for barley and bean, respectively. For light-adapted leaves, the decrease was characterized by u_0.5_-values of 0.52 m s^-1^ for barley and 0.33 m s^-1^ for bean. Additionally, τ-values of light-adapted leaves were significantly lower compared to dark-adapted leaves (p < 0.05 for barley and p < 0.001 for bean). Significant differences in τ between barley and bean were found at wind speeds of 0.0 m s^-1^ and 0.8 m s^-1^ (p < 0.05). Absolute τ-values were always higher for bean leaves compared to barley leaves irrespectively of the wind speed and adaptation state. At zero wind, the mean τ was more than 10 s higher for bean compared to barley.

We could not observe a comparable pattern in the response of T_L_-T_a_ to both wind and light (**Figures 2C, D**). For barley leaves, T_L_-T_a_ seemed to remain relatively stable throughout the measurements (p > 0.05), while light-adapted leaves were generally cooler compared to dark-adapted leaves. Significant differences between dark- and light-adapted leaves, however, were only found at wind speeds of 0.0 m s^-1^ and 0.4 m s^-1^ (p < 0.05). For dark-adapted bean leaves we observed an exponential decrease in T_L_-T_a_ in response to increasing wind speed, and an exponential increase in response to increasing wind speed for light-adapted leaves (**Figure 2D**). At zero wind, light-adapted leaves were cooler compared to dark-adapted leaves. At a wind speed of 0.4 m s^-1^, T_L_-T_a_ values were similar and when wind speeds exceeded 0.4 m s^-1^ light-adapted leaves were warmer compared to dark-adapted leaves. Except at wind speeds of 0.4 m s^-1^ the differences between dark- and light-adapted leaves were significant (p < 0.05).

For both barley and bean, gs increased significantly (p < 0.05) in response to light (**Figures 2E, F**). Although g_s_ slightly decreased on average in response to increasing wind speed, we did not find any significant changes, neither for barley, nor for bean.

### Wind- and Light Induced Spatial Variations in T_L_-T_a_

**Figure 3:**
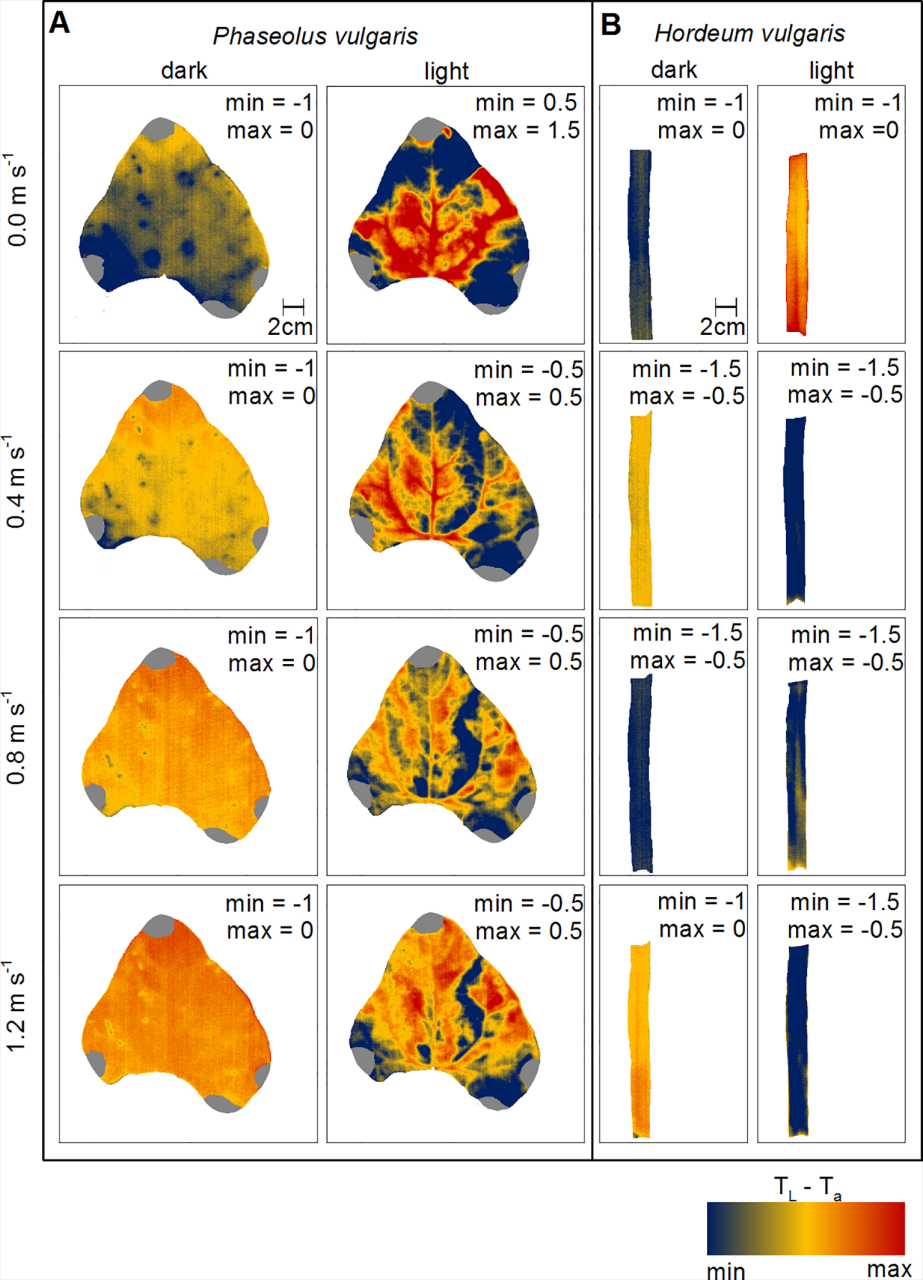
Spatial mapping of wind- and light-induced changes in leaf temperature to ambient air temperature difference (T_L_-T_a_). **(A)** Representative leaf of common bean (Phaseolus vulgaris) and **(B)** representative leaf of spring barley (Hordeum vulgare). Dark-adapted leaves are presented in the first column from the left and light-adapted leaves in the second column from the left, respectively. Each row represents measurements at different wind speeds of 0.0 m s^-1^, 0.4 m s^-1^, 0.8 m s^-1^, and 1.2 m s^-1^. T_L_-T_a_ are color-coded as indicated by the false-colorscale at the bottom. Minimum (min) and maximum (max) values of T_L_-T_a_ are given in each panel.

To evaluate changes in leaf heat transfer in response to wind and light illumination we mapped T_L_-T_a_ spatially for representative leaves of bean and barley, respectively (**Figure 3**). Generally, dark-adapted leaves had a more homogeneous distribution of T_L_-T_a_ over the leaf surface compared to light-adapted leaves (**Figure S8**). We observed that T_L_-T_a_ for dark-adapted leaves appeared to become more homogeneous with increasing wind speed from 0.0 to 1.2 m s^-1^. For light-adapted bean leaves, areas in between major veins generally appeared cooler compared to areas with a comparably higher density of major veins. Particularly, at zero wind, leaf areas where lower order veins are located and areas which are nearer to the leaf edges were cooler than the center of the leaf. With increasing wind speed, T_L_-T_a_ increased and areas that appeared cooler earlier got warmer, particularly at the leaf tip, which was in the direction of the wind-leading edge. For barley leaves, the observed patterns were not as clear as those observed for bean leaves (**Figure 3B**). For example, when leaves were light-adapted at wind speeds of 0.0 m s^-1^ and 0.8 m s^-1^, the mid-vein was only partly visible because it was warmer compared to the leaf lamina.

### Wind- and Light Induced Spatial Variations in τ

**Figure 4:**
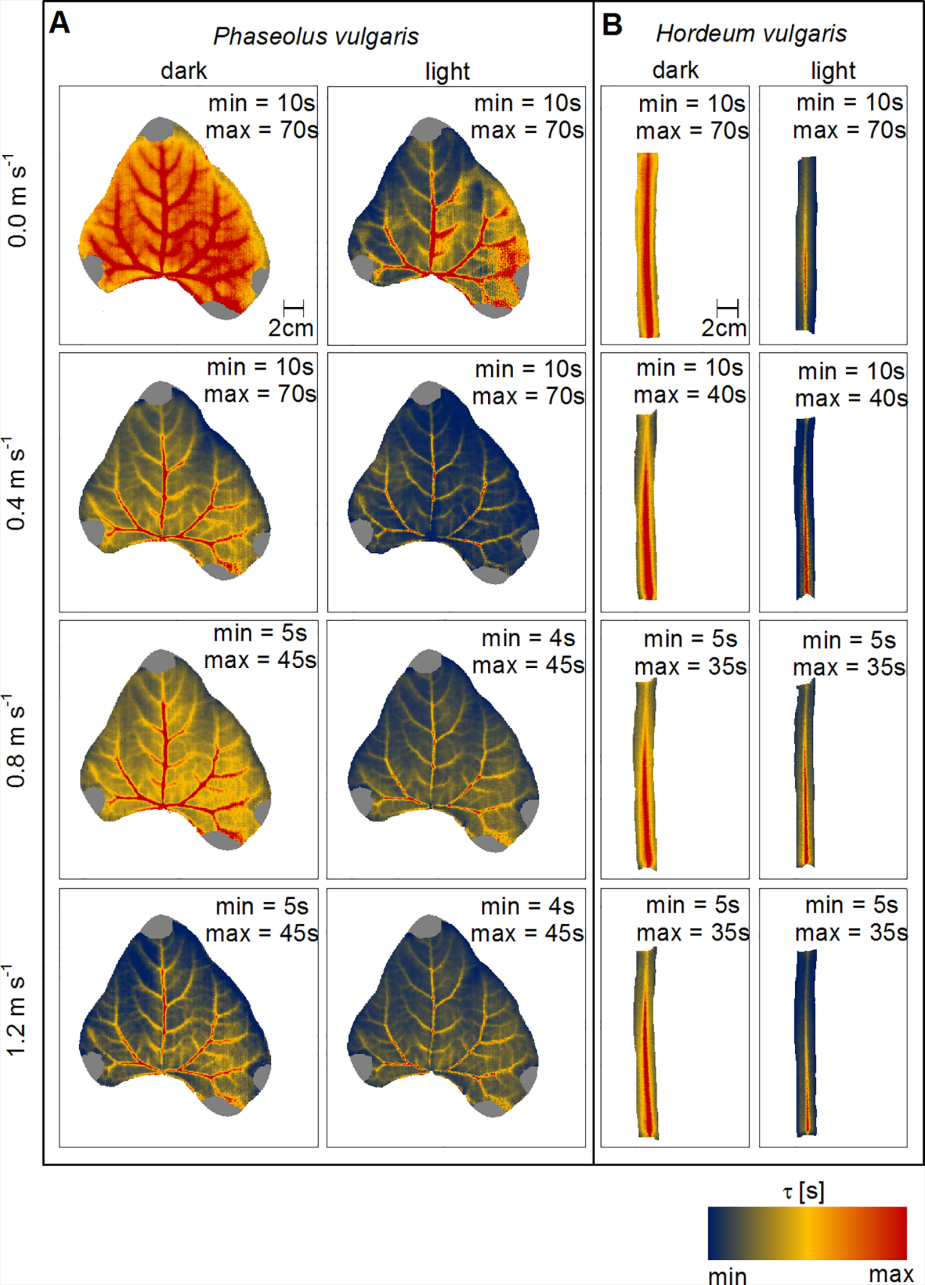
Spatial mapping of wind- and light-induced changes in time constant (τ). **(A)** Representative leaf of common bean (Phaseolus vulgaris) and **(B)** representative leaf of spring barley (Hordeum vulgare). Dark-adapted leaves are presented in the first column from the left and light-adapted leaves in the second column from the left, respectively. Each row represents measurements at different wind speeds of 0.0 m s^-1^, 0.4 m s^-1^, 0.8 m s^-1^, and 1.2 m s^-1^. T_L_-T_a_ are color-coded as indicated by the false-color scale at the bottom. Minimum (min) and maximum (max) values of τ are given in each panel.

Spatial maps of τ provide information on the thermal responsiveness of a leaf, because this quantity is related to both, water distribution in the leaves and h_leaf_. As we observed for the mean values, the images illustrate that with increasing wind speed and with light-illumination τ decreased (**Figure 4**). The most prominent structures were the leaf vascular tissues, which was reflected by comparably higher τ-values irrespective of wind speed and illumination state. While we were able to detect second and third order veins on the bean leaf (**Figure 4A**), we could only map the main-vein on the barley leaf (**Figure 4B**). In dark-adapted leaves, the highest τ-values were associated with larger order veins near the leaf base. Additionally, a gradient from high to low τ-values could be observed from the leaf base to the leaf tip and towards the leaf edges (**Figure S9**). Generally, in response to light τ decreased. However, the most prominent leaf structures were the veins, as indicated by higher τ-values compared to the leaf blades, where smaller order veins are located. Still, major, second and third order veins were visible in bean leaves.

We analyzed the image pixels for nonlinear correlations using the Spearman correlation analysis and found a significant correlation between T_L_-T_a_ and τ for barley and bean at all experimental levels (p < 0.05).

### Correlation Between Modeled h_leaf_ and τ

**Figure 5:**
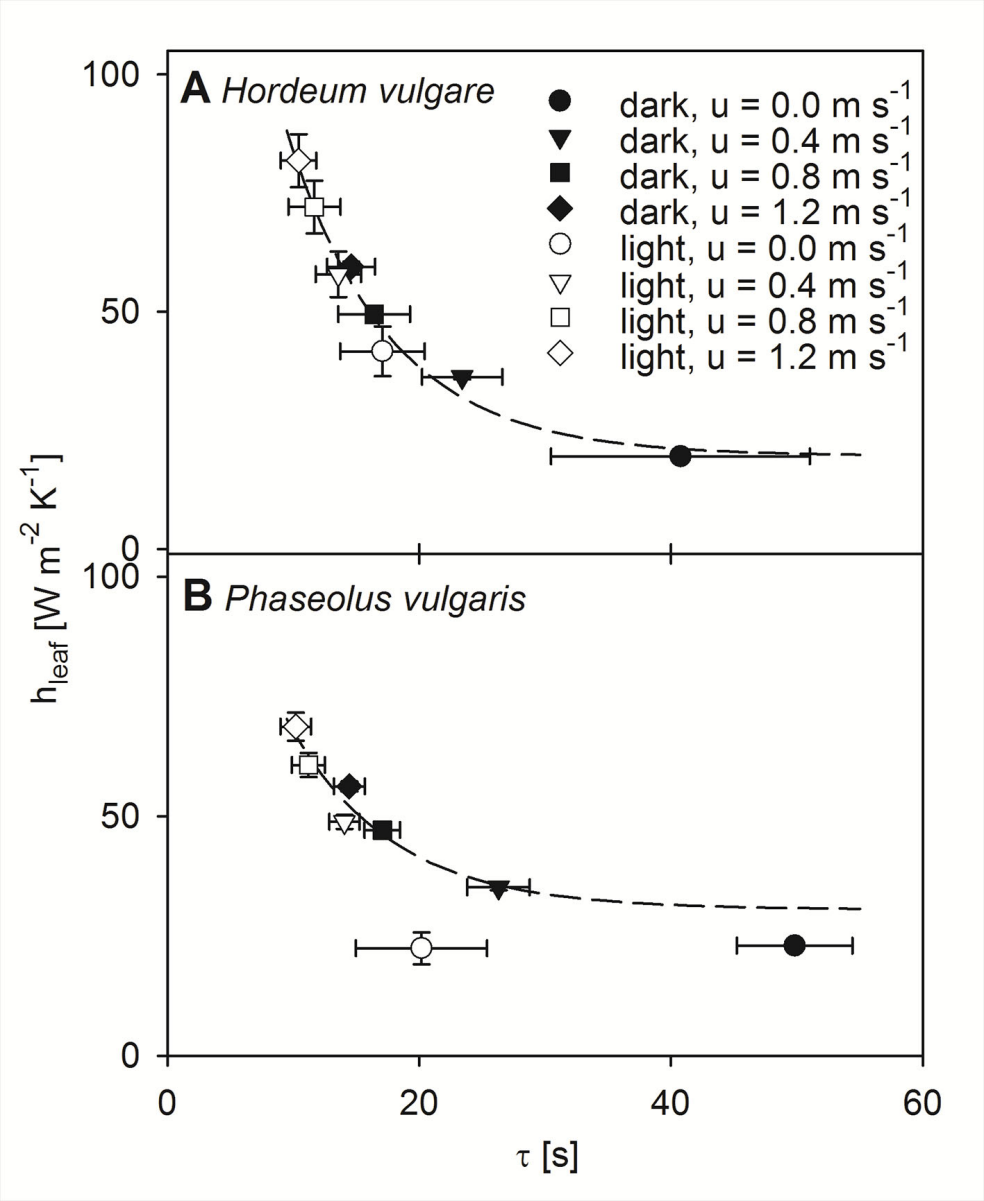
Correlation between modeled leaf heat transfer coefficient (hleaf) and time constant (τ). Measurements were performed at four different wind speeds and in dark- (closed symbols) and light-adapted (open symbols) state of individual leaves. Measurement at a wind speeds of 0.0 m s^-1^ shown by circles, 0.4 m s^-1^ shown by triangles, 0.8 m s^-1^ shown by squares, and 1.2 m s^-1^ shown by diamonds. Dark-adapted leaves are indicated by closed symbols and light-adapted leaves are indicated by open symbols. **(A)** Measurements of individual spring barley leaves (Hordeum vulgare). Exponential regression indicated by dashed line: f(x) = 19.60 + 221.81 e^-0.12x^, r² = 0.97. **(B)** Measurements of individual common bean leaves (Phaseolus vulgaris). Exponential regression are indicated by a dashed line: f(x) = 30.56 + 128.29 e-0.12x, r² = 0.89. Error bars represent standard deviation of single leaves. For barley n = 9, and for bean n = 10.

Using the dimensionless numbers approach we calculated h_leaf_ for each experimental level (**Figure 5**). Note here that, because the modeled data for bean leaves at free convection revealed some weaknesses most likely due to a weak linear relationship between τ and LWC (**Figure 1B**), these data were consequently excluded from further statistical analyses. For both barley and bean, we found a highly significant correlation between the modeled h_leaf_ and the measured τ (p < 0.001). Generally, h_leaf_ of light-adapted leaves were higher compared to h_leaf_ of dark-adapted leaves. Relationships between h_leaf_ and τ were characterized by exponential regressions, which revealed the time that is required to reach 50% of the initial value (t_0.5_). For both plant species t_0.5_-values of 5.6 s were obtained, indicating a similar response of τ to h_leaf_. However, absolute h_leaf_-values were higher for barley leaves, compared to bean leaves. While barley leaves reached on average maximum values of 81.8 (± 5.6) W m^-2^ K^-1^ (**Figure 5A**), bean leaves reached on average maximum values of 68.7 (± 2.9) W m^-2^ K^-1^ (**Figure 5B**).

### Contribution of h_LW_, h_H_, and h_λE_ to the Overall h_leaf_

**Figure 6:**
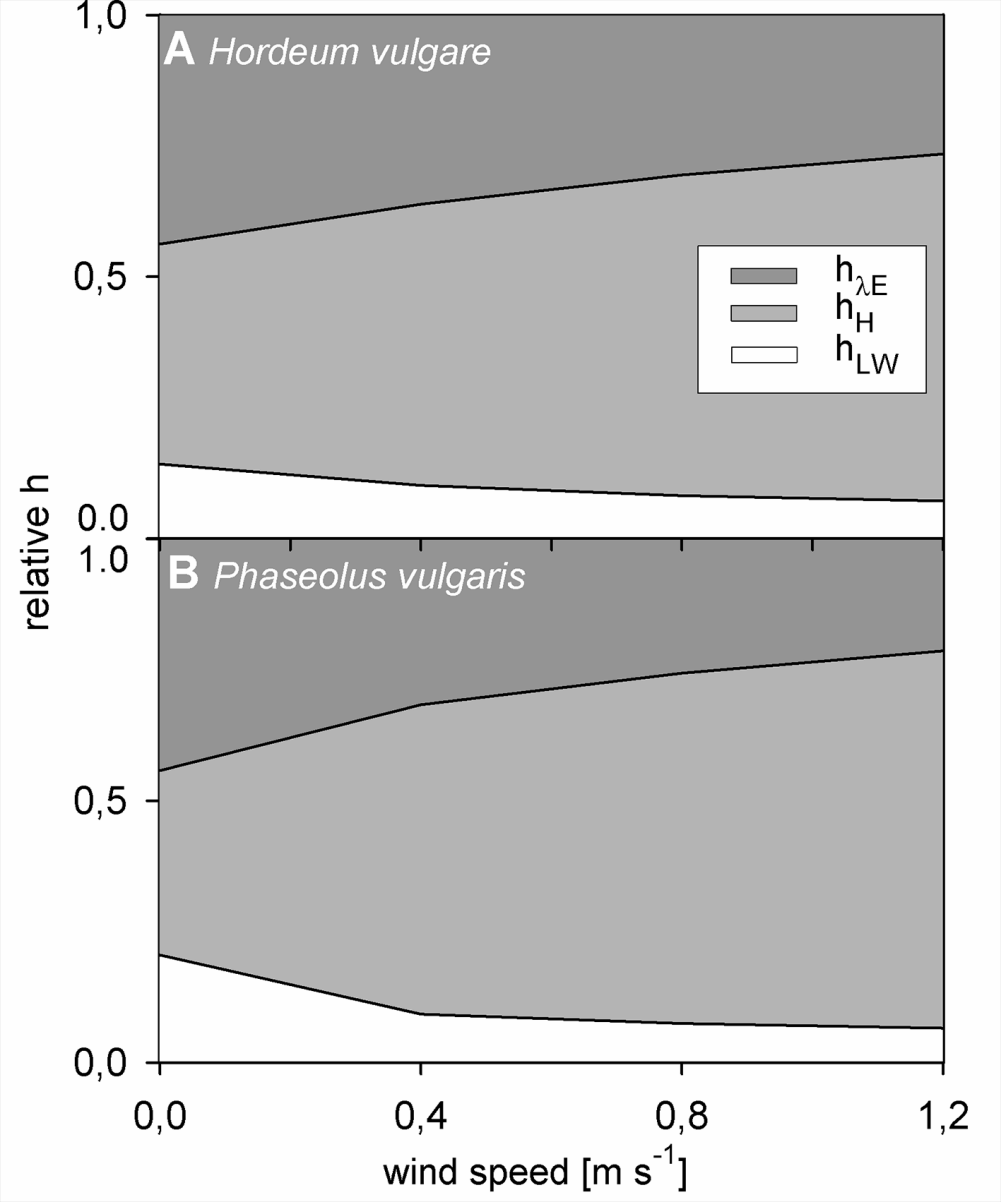
Relative contribution of normalized heat transfer coefficients to the total leaf heat transfer coefficient in response to wind speed of light-adapted leaves. Heat transfer coefficient for evapotranspiration heat (h_λE_) in dark grez, heat transfer coefficient for convective heat (h_H_) in light grey, and heat transfer coefficient for long-wave radiative heat (h_LW_) in white. **(A)** Spring barley (Hordeum vulgare) and **(B)** common bean (Phaseouls vulgaris). Areas represent mean values of n = 9 single leaves for barley and n = 10 single leaves for bean.

Finally, we evaluated the impact of each heat transfer coefficient on the overall h_leaf_ on light-adapted leaves. For this evaluation, the portion of the respective heat transfer coefficient in the entire h_leaf_ was calculated (e.g., h_H_ h_leaf_^-1^). While hLW did not correlate with hleaf, for both barley and bean, hH and hλE showed a strong and significant correlation with hleaf (p < 0.001). The relative contribution of each heat transfer coefficient to the overall hleaf significantly changed with wind speed (p < 0.001) (**Figure 6**). While the relative contribution of hH increased with increasing wind speed, the relative contribution of hLW and hλE decreased with increasing wind speed. At zero wind hλE had the highest impact on hleaf accounting for about 45% of the overall hleaf. However, albeit lower, at 1.2 m s^-1^ the relative contribution of hλE was still significant with a contribution of 26% and 21% for barley and bean, respectively.

## Discussion

In this paper, we introduce the active thermography approach as a powerful method to evaluate leaf heat transfer processes of intact leaves. We thoroughly tested the active thermography experimental protocol in the laboratory on spring barley and common bean leaves, by comparing measured τ to modeled hleaf. We found strong relationships between τ and hleaf, which were valid for dark- and light-adapted leaves at varied wind speeds. Additionally, spatial T_L_-T_a_- and τ-maps revealed the impact of local differences in thermal responsiveness, related to C A^-1^_leaf_ and h_leaf_ differences, on T_L_.

To evaluate the measured responses of τ with respect to changes in h_leaf_, we modeled h_leaf_ using dimensionless numbers. We evaluated the modeled data by comparing C A^-1^_leaf_ derived from dimensionless numbers and τ introducing C A^-1^_leaf_ derived from the linear relationships found between τ and LWC (**Figures 1**, **S2**, and **S5**).

These models yielded comparable values with some differences at zero wind, particularly for bean. If no wind is present, free convection dominates, which mainly depends on leaf area and surface structure (compare Eq. S3). Because the variation in leaf area was much higher for bean leaves (± 46 cm²) than for barley leaves (± 5.5 cm²), we assume a high variability of hleaf leading to errors in the linear relationship between τ and LWC. Both measurements and the modeling of heat transfer in wind-free conditions assuming free convection are difficult, because leaf area and rough surface elements affect the heat transfer more strongly than during forced convection (Kumar and Barthakur, 1971). Particularly for bean leaves, we could observe great spatial heterogeneity of T_L_-T_a_ over single leaves similar to prior simulations (Roth-Nebelsick, 2001) and that may result in thermal instabilities of the boundary layer (Defraeye et al., 2013). In addition to leaf area, surface roughness, caused by trichomes and vascular tissue, affects hleaf (Parkhurst, 1976; Schreuder et al., 2001). In contrast to bean, barley has a relatively flat surface with a regularly arranged and parallel vein system (Dannenhoffer et al., 1990; Ueno et al., 2006). Bean has an uneven surface with plenty of thick veins that are dichotomously branched, which is likely to disturb the air movements across the leaf surface and influence heat transfer. Furthermore, heterogenous T_L_-T_a_ may be a result of heterogeneous vein density and distribution of stomata over the leaf surface, which both affect hleaf. It was observed that the leaf conductance to water vapor was up to 18% higher at the leaf tips compared to the leaf base, which was explained by a higher vein density in this region (Nardini et al., 2008). Particularly in free convection conditions and at low wind speeds, leaf tips appeared to be cooler compared to the leaf base (**Figure 3**), which might be attributable to comparable higher leaf conductance to water vapor. Heterogeneity in the density and distribution of stomata over the leaf surface (Fanourakis et al., 2015), may affect T_L_-T_a_ in a similar way.

Our observation that measured τ and modeled hleaf decrease in response to increased wind-speed, which affects the boundary layer thickness, is in agreement with previous findings (Raschke, 1960; Vogel, 2009). Generally, barley leaves showed higher hleaf and lower τ-values compared to bean leaves. Smaller and narrower leaves have a thinner and more homogeneous boundary layer (Gates, 1965; Sinclair, 1970; Roth-Nebelsick, 2001). However, the response to wind was quite similar for barley and bean leaves, as indicated by u_0.5_-values (**Figure 2**). If wind is present, the leaf boundary layer thickness will be reduced and heat will be increasingly transported away from the leaf surface with the air movement, which increases hleaf and thus decreases τ (Schuepp, 1993; Vogel, 2009; Defraeye et al., 2013). The assumption that the boundary layer thickness is progressively reduced (Kitano and Eguchi, 1990) is supported by spatial T_L_-T_a_ and τ-maps of bean leaves. In our experiments the wind-leading edge (leaf tip) in response to wind got warmer and τ decreased compared to the wind-averted leaf edge. For barley this effect was not visible, which might be related to generally more homogeneous boundary layer and smoother leaf surface that offers less resistance to the air stream (Gates, 1965; Sinclair, 1970; Dannenhoffer et al., 1990).

We have observed a decrease in τ of about 24 s and 34 s upon illumination for barley and bean, respectively (**Figures 2A, B**). Based on the established linear relationships (**Figure 1**), these changes would correspond to a water loss of 20% to 30%, so that the decrease in τ cannot be explained by a decrease in LWC. However, light induces stomatal opening, which increases hleaf and particularly h_λE_, (Mott et al., 1997; Roelfsema and Hedrich, 2005; Shimazaki et al., 2007; Pieruschka et al., 2010). In our experiments, we found that h_λE_ contributes about 45% to the overall hleaf for light-adapted leaves under wind free conditions. Thus, τ is strongly affected by leaf conductance to water vapor, although the relative contribution of h_λE_ to the overall hleaf decreased with increasing wind speed. At higher wind speeds, stomatal resistance has larger effect on water vapor fluxes than the convective resistance (Cannon et al., 1979; Defraeye et al., 2013). Consequently, h_λE_, which depends on both convective and stomatal resistance, increases less strongly in response to increased wind compared with h_H_, which depends on convective resistance alone. Thus, under these conditions the relative contribution of h_λE_ to the overall hleaf decreases whereas the relative contribution of hH increases. Nevertheless, because both barley and bean leaves were transpiring at high rates (barley: 0.35 (± 0.03) mol m^-2^ s^-1^, bean: 0.22 (± 0.02) mol m^-2^ s^-1^), some water loss is also very likely, which results in an even stronger decrease of τ.

To evaluate the impact of τ on T_L_, and thus the thermal responsiveness on T_L_, we mapped both, τ and T_L_-T_a_ spatially (**Figures 3** and **4**). Correlations between T_L_-T_a_ and τ in our experiments indicate a strong relationship between T_L_ and thermal responsiveness. The highest τ-values were associated with leaf vascular tissues, because leaf veins have a higher C A^-1^_leaf_ compared to the leaf lamina (McKown et al., 2010; Sack and Scoffoni, 2013). Particularly visible at bean leaves, those areas, which were associated with higher τ-values, appeared also warmer compared to the rest of the leaf. Near the veins the density of stomata is low and with it the leaf conductance to water vapor and thus the overall leaf heat transfer. In contrast, areas in between the larger order veins, where the stomata density is higher and with it the leaf conductance to water vapor, showed lower τ-values and appeared cooler. Additionally, in these regions leaf thickness is lower and less water is present. We conclude that local variations in the leaf heat transfer and water within the leaves (≈ C A^-1^_leaf_) will result in local differences of thermal responsiveness of the leaf and thus T_L_. Using the computed spatial maps of τ, it is possible to separate areas in which thermal responsiveness mainly depends on C A^-1^_leaf_ from those in which thermal responsiveness mainly depends on hleaf. Finally, those τ-maps may contribute to studies aiming at detect heterogenous leaf conductance to water vapor (e.g., Nardini et al., 2008) or at detection of stomata heterogeneity (e.g., Fanourakis et al., 2015).

Our results indicate that h_H_ is essential to assess the overall h_leaf_. At all experimental conditions h_H_ strongly contributed to h_leaf_ (**Figure 6**). Only at zero wind speed hleaf was dominated by h_λE_. At all remaining conditions, h_H_ was the dominant heat transfer coefficient. Convective heat and transpiration are inevitably coupled because heat associated with water vapor must penetrate the boundary layer depending on the prevailing convective conditions. It follows that modeling of transpiration requires consideration of convective processes. Because τ is a good measurement of h_leaf_ as demonstrated here and in agreement with previous reports (Pearman, 1972; Parlange and Waggoner, 1971; Saldin and Barthakur, 1971; Kumar and Barthakur, 1971) and we could also map τ spatially using active thermography, this approach should contribute to more precise modeling of leaf heat transfer processes in the future.

Plant water relations and h_leaf_, both related to τ, are of importance for modeling plant-environment interactions (Foley et al., 2003), as well as for plant phenotyping, especially considering the necessary gain in plant productivity and water-use-efficiency under water-limited agriculture (Passioura and Angus, 2010; Sampoux et al., 2011; Farrar et al., 2011). We provide experimental evidence that τ measured with the active thermography approach is an important parameter describing leaf heat transfer processes.

However, we also want to point out the novelty of our approach, as active thermography is not well established in plant sciences yet. Unidentified sources of error may still persist, as for instance uncertainties in model parameterization. While the values for the numeric constant b for free and mixed convection (cf. Eqs. 7 and 8, **Table S1**) were in the range of values presented by Dixon and Grace (1983), values for the constant a were considerably higher than reported previously for artificial leaves in a laminar air flow (Bailey & Meneses, 1993). Additionally, we found different values for a under conditions of free and mixed convection, whereas Bailey and Meneses (1993) reported this not to be the case. The question arises if the differences in experimental setups (e.g., artificial leaves vs. plant leaves, laminar air flow vs. turbulent air flow) allow a direct cross-comparison of the numeric constants. The application of active thermography requires constant ambient conditions during the induced T_L_-shift and the subsequent cooling process, and consequently this approach is only applicable in (semi)-controlled conditions. As mentioned above, we measured a small increase of background temperature after application of the heat pulse (0.3–0.4 K). However, background temperature remained stable during the subsequent cooling phase. Thus, we conclude that ambient conditions were kept sufficiently stable to allow reliable and repeatable measurements. Still, we recommend the use of an alternative heat source for future experiments. Especially shorter response times of the IR heating units would be desirable. Newly developed IR-LED panels may provide an improvement here.

In this study, we deliberately chose a realistic setup resembling natural conditions, relating active thermography to independent parameters, as for instance leaf transpiration. For further validation of our parameterization, we aimed to test our setup under more standardized conditions, including artificial reference materials, also helping us to calibrate our experimental setup. Measurements using differently wetted sheets of filter paper (**Figure S10**) confirmed the strong dependency of leaf cooling curves on the water content per area, as presented in **Figure 1**. However, there is no reference material available to mimic the highly variable structural and physiological properties of plant leaves entirely.

We conclude that active thermography provides a powerful tool in studying plant water relations and plant heat transfer processes when ambient conditions are monitored simultaneously and in high detail. The adaptability of the method towards semi-controlled conditions opens the way to new applications of active thermography in plant sciences, as for example in large-scale green-house phenotyping facilities.

## Author Contributions

HA: designed and performed the experiments and analyzed the data, conceived the project and wrote the article with contributions of all authors. FF: supervised the experiments, supervised and complemented the writing. RP: supervised the experiments, supervised and complemented the writing. MM-L: analyzed the data and complemented the writing. CJ: complemented the writing, designed and performed further experiments and analyzed the data. LS: supervised and complemented the writing. US: supervised and complemented the writing. UR: supervised the experiments, supervised and complemented the writing.

## Funding

The Helmholtz Association institutionally funds research at IBG-2 Plant Sciences (POF III Program - Research Field Key Technologies – Key Technologies for the Bioeconomy). Part of this work was performed within the German-Plant-Phenotyping Network (DPPN) which is funded by the German Federal Ministry of Education and Research (project number: 031A053). The authors also acknowledge the funding of the PhenoCrops project in the context of the Ziel 2 – Programm NRW 2007-2013 “Regionale Wettbewerbsfähigkeit und Beschäftigung” by the Ministry for Innovation, Science and Research (MIWF) of the state North Rhine Westfalia (NRW) and European Union Funds for regional development (EFRE) (005-1105-0035).

## Conflict of Interest

The authors declare that the research was conducted in the absence of any commercial or financial relationships that could be construed as a potential conflict of interest.
