# Supplementary material for: Quantitative Estimation of Leaf Heat Transfer Coefficients by Active Thermography at Varying Boundary Layer Conditions
Source: Front Plant Sci. 2020 Jan 21;10:1684. doi: 10.3389/fpls.2019.01684 (PMC6985100; doi:10.3389/fpls.2019.01684)
Supplement: Supplementary file 1 [file DataSheet_1.pdf]

Supplementary Material

Model description

According to equation 4, calculation of h_{leaf} requires the parameters τ and C A^{-1}_{leaf}. To derive those parameters for the actual measurements, two series of reference measurements on dark-adapted plants were performed.

In the first reference measurements, we established the relationship between τ and leaf water content per unit area (LWC) (Fig. 1). To obtain C A^{-1}_{leaf} LWC was multiplied with the specific heat capacity of water (4200 J kg^{-1} K^{-1}). Because the relationships between τ and LWC were obtained at only three wind speeds, we defined a wind speed dependent linear equation to model more broadly LWC leaves exposed to different wind speeds.

$$LWC = f_m(u)\tau + f_y(u) \qquad \text{Eq. S1}$$

The slope of the linear equation is given by the exponential function $f_m(u)$ and the y-intercept is given by the exponential function $f_y(u)$. Both the relationships and the respective exponential equations are given in figure S1.

To model h_{leaf} according Eq.5 to Eq.8, the numerical constants a and b (see Eq. 8), which are related to the leaf geometry, are required to estimate the heat transfer coefficient for convective heat (h_H). There are several values available in literature for example given by Monteith and Unsworth (2008) or Dixon and Grace (1983). However, in order to achieve a possibly accurate estimation of h_{leaf} valid for our experimental conditions, we experimentally determined these parameters on a separate set of dark-adapted plants.

First, we had to model h_H and the respective conductance to convective heat (g_H), which cannot be easily measured directly. For this purpose, we used dimensionless numbers, which relate the mean leaf diameter (d) to the boundary layer thickness. This ratio is known as Nusselt number

(Nu) (e.g. Monteith and Unsworth, 2008; Nobel, 2009). Because, g_H is a function of leaf diameter and boundary layer thickness, Nu can be used for calculations of g_H with respect to leaf properties, such as size and shape. Using Nu, g_H may be calculated as follows (Dixon and Grace, 1983):

$$g_H = \frac{Nu\,k}{\rho_a c_p d} \qquad \text{Eq. S2}$$

Here, k is the thermal conductivity of air (0.024 W m^{-1} K^{-1}), ρ_a the density of air (1.204 kg m^{-3}), and c_p the specific heat capacity of air (1005 J kg^{-1} K^{-1}).

In free convection, the heat transfer depends on upwelling air movement from the leaf surface into ambient air, which is maintained by the difference between leaf temperature and ambient air temperature (T_L-T_a). Under these conditions, Nu is the function of another dimensionless number, the Grashof number (Gr). Using Gr, the boundary layer is mainly characterized by T_L-T_a, the leaf dimension, and buoyancy forces of air. The detailed calculation of Gr is as follows:

$$Gr = \frac{g\,\beta d^3 (T_L - T_a)}{\nu^2} \qquad \text{Eq. S3}$$

Here, g is the gravitational acceleration (9.81 m s^{-2}), β the thermal expansion coefficient of air (0.0034 K^{-1}), and ν the kinematic viscosity of air (15.6 * 10^{-6} m^2 s^{-1}).

With Gr, Nu becomes:

$$Nu = aGr^b \qquad \text{Eq. S4}$$

Here, a and b are the numerical constants describing the geometry of a leaf.

For forced convection, conditions where wind occurs, Nu becomes a function of the Reynold's number (Re). Re relates the boundary layer composition to the wind speed (u) and d of a leaf.

$$Re = \frac{u\,d}{\nu} \qquad \text{Eq. S5}$$

Similar to the formulation for free convection in forced convection Nu becomes:

$$Nu = aRe^b \qquad \text{Eq. S6}$$

We have used moderate wind treatments in our experiments. Because at low wind speeds free convection still occurs, it is convenient to use mixed convection to describe convective heat

transfer. This implies both free and forced convection contributes to leaf heat transfer. In our study, the following formulation of the mixed convection was used (Bailey, 1993):

$$Nu = a(Gr + 1.4Re^2)^b \qquad \text{Eq. S7}$$

To calculate g_H according to equation S2, the parameters a and b are required, which we derived from our reference measurements, where dark-adapted leaves were exposed to step-wise increasing wind-speeds from 0 m s^{-1} to 1.2 m s^{-1}.

In a first step, we calculated g_H of the dark-adapted leaves by rearranging Eq. 4 and making two assumptions:

1. The heat transfer coefficient for evapotranspiration ($h_{\lambda E}$) can be neglected for dark-adapted leaves. This assumption is supported by the measured values, i.e. g_s was very low for barley and bean leaves in the dark (Fig. 1).
2. g_H behaves in a similar way irrespective of dark- or light-adaptation.

Using these assumptions, g_H could be calculated as follows:

$$g_H = \left(\frac{\frac{C}{A_{leaf}}}{\tau} - h_{LW}\right)\left(\frac{1}{\rho_a c_p}\right) \qquad \text{Eq. S8}$$

To calculate g_H according to Eq. S8 we first derived C A_{leaf}^{-1} using the model from the first series of reference measurements, where the relationship between τ and LWC was established. In a second step, we used the calculated g_H to calculate the respective Nu (Eq. S2). This calculated Nu was compared to theoretical Nu values, obtained by equation S7 with the variables a and b being 1. By plotting the logarithm of the measured Nu (log $Nu_{measured}$) against the logarithm of equation S7 ($\log(Gr + 1.4\ Re^2)$), we obtained both parameters, a and b (Fig. S2). Note here that Eq. S7 equals Eq. S4 for leaves exposed to free convection conditions. The respective values for barley and bean and for free and mixed convection are given in table S1.

Table S1: Parameters a and b for spring barley (*Hordeum vulgare*) and common bean (*Phaseolus vulagris*) leaves at free and mixed convection.

	Hordeum vulgare		*Phaseolus vulgaris*	
	a	b	a	b
free convection	13.76	0.16	0.91	0.33
mixed convection	2.41	0.25	2.58	0.25

By substituting a and b into equation S7, g_H could be calculated at any prevailing conditions. Figure S3 summarizes the workflow of the whole modelling approach and the final experiment. We tested the dimensionless numbers model by comparing calculated C A^{-1}_{leaf} based on the linear relationships (Fig. S1) with C A^{-1}_{leaf} based on dimensionless numbers (Fig. S4). Generally, both models matched well to each other. For barley, we found a strong linear correlation, which was highly significant ($p < 0.001$ and $r = 0.97$). In contrast, the model using dimensionless numbers revealed some weaknesses for bean, particularly for C A^{-1}_{leaf} calculations for non-wind conditions (Fig. 5.4b). Here the dimensionless numbers model clearly underestimates C A^{-1}_{leaf} compared to the model based on the linear regressions. Because the models did not match very well for 0.0 m s^{-1}, these values were excluded from further statistical analyses. However, for the wind speeds between 0.2 and 1.2 m s^{-1} we found a strong and highly significant linear correlation between the two models also for bean ($p < 0.001$ and $r = 0.986$).

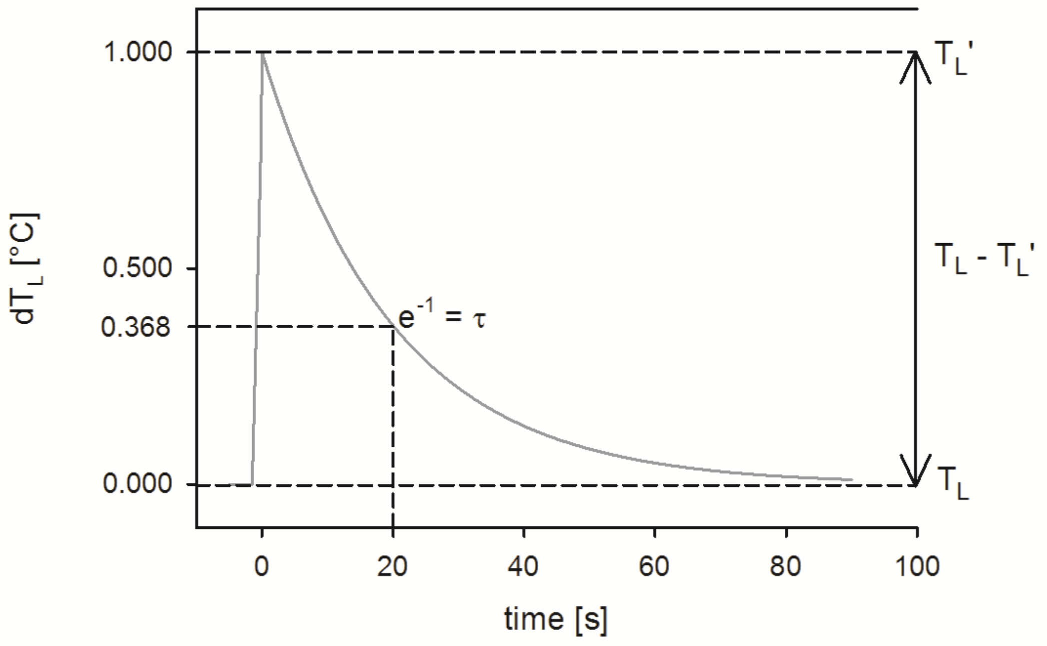

Figure S1: Principle of active thermography. A heat pulse was applied to a leaf, inducing a shift from steady state leaf temperature (T_L) to a transient T_L (T_L'). Leaf cooling kinetic is characterized by an exponential decay constant (e^{-1}), which is the time constant (τ) and is the time required to reach about 36.8% of T_L-T_L'.

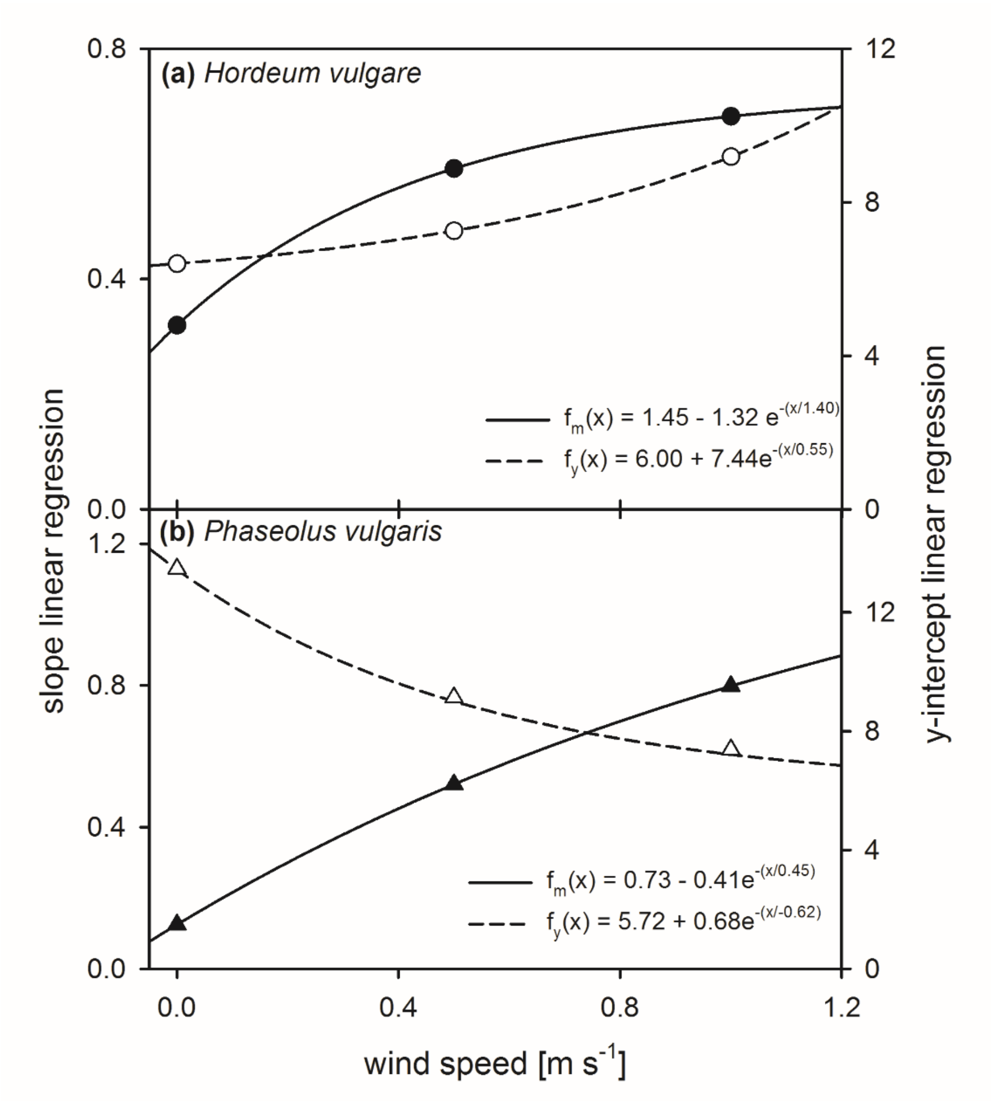

Figure S2: Linear regression model for leaf water content prediction. The slope (m) obtained by linear regression of the relationship between the time constant and leaf water content is given by closed symbols and the y-intercept of the linear regressions is given by open symbols. Change of the regression parameter in response to wind speed were fitted using exponential regressions. Equations are given in the respective panel.

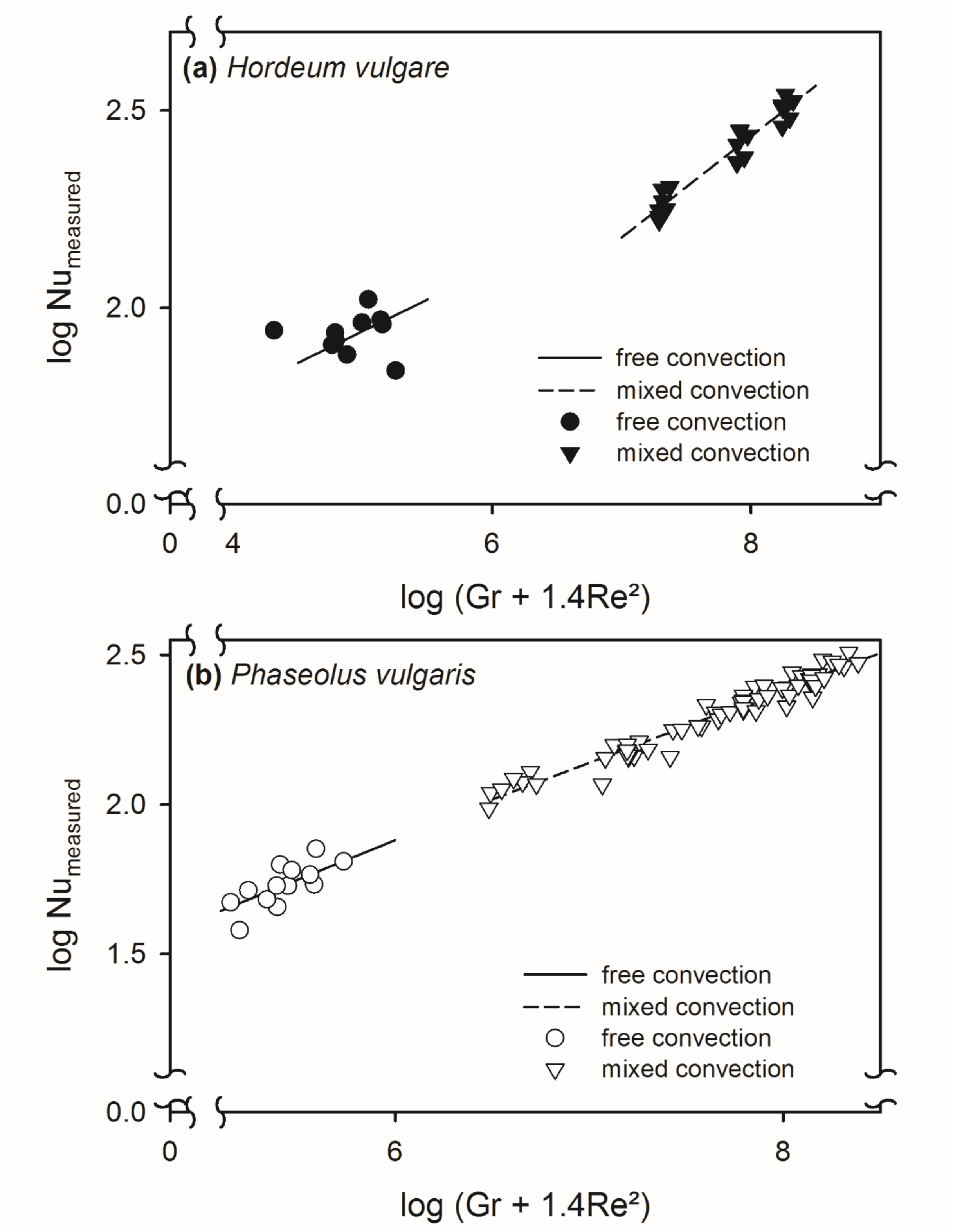

Figure S3: Derivation of the parameters a and b for the dimensionless numbers model. Logarithm of the Nusselt number (Nu) derived from measured data was plotted against the logarithm of the theoretical Nu, given by Gr + $1.4Re^2$. **(a)** Measurements of dark-adapted spring barley leaves (*Hordeum vulgare*)

under free convection conditions (circles) and mixed convection conditions (triangles). Two linear regressions were applied. Free convection indicated by solid line with $y = 0.16x + 1.14$, $r^2 = 0.95$, mixed convection (dashed line) with $y= 0.26x + 0.38$, $r^2 = 0.93$. **(b)** Measurements of dark-adapted common bean leaves (*Phaseolus vulgaris*) under free convection (circles) and under mixed convection conditions (triangles). Two linear regressions were applied. For free convection (solid line): $y= 0.26x + 0.30$, $r^2 = 0.75$, and for mixed convection (dashed line): $y=0.24y + 0.43$, $r^2 = 0.95$.

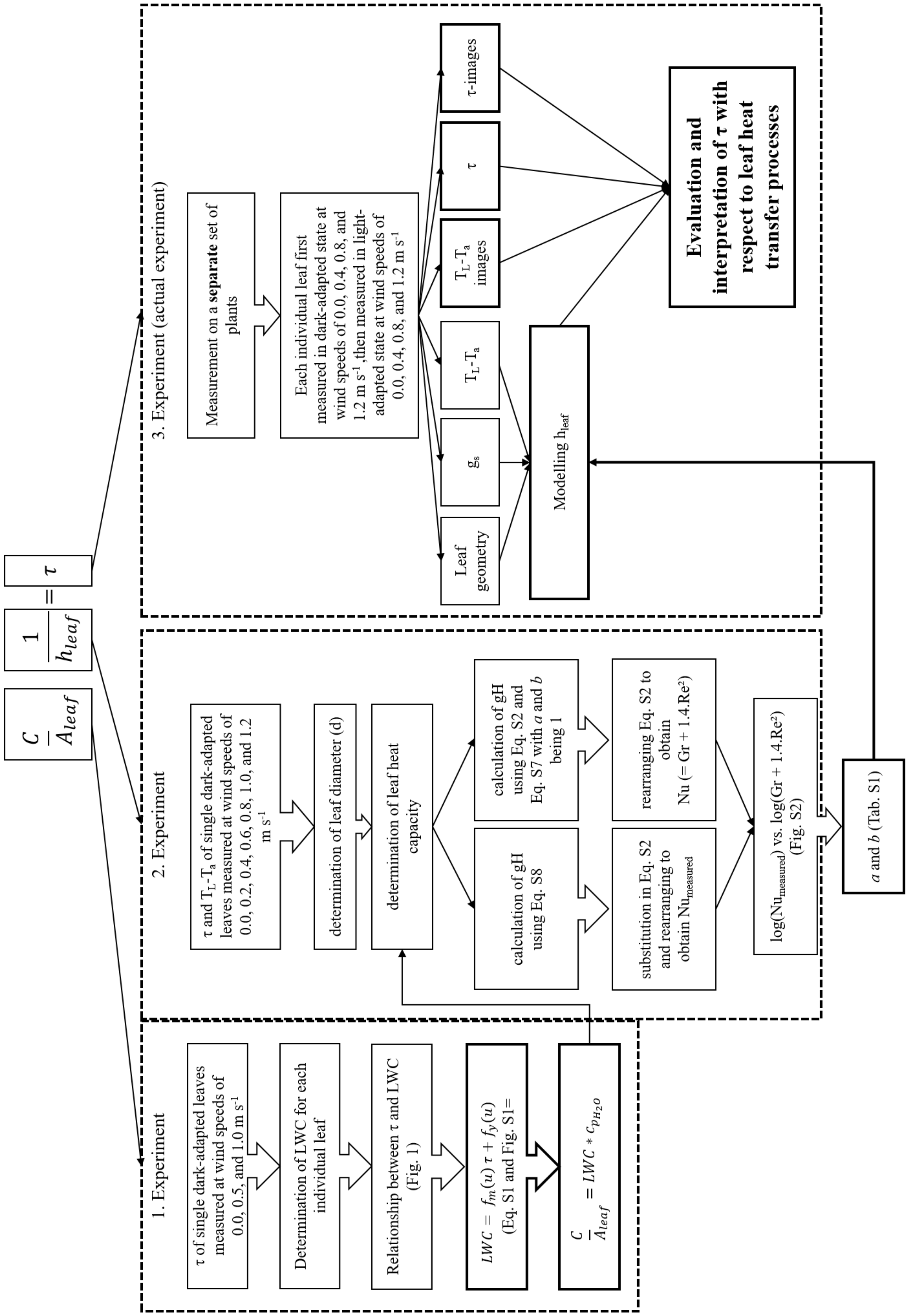

$\frac{C}{A_{leaf}}$ $\frac{1}{h_{leaf}}$ $= \tau$
1. Experiment
τ of single dark-adapted leaves measured at wind speeds of 0.0, 0.5, and 1.0 m s-1
Determination of LWC for each individual leaf
Relationship between τ and LWC (Fig. 1)
$LWC = f_m(u)\,\tau + f_y(u)$ (Eq. S1 and Fig. S1=
$\frac{C}{A_{leaf}} = LWC * c_{p_{H_2O}}$
2. Experiment
τ and TL-Ta of single dark-adapted leaves measured at wind speeds of 0.0, 0.2, 0.4, 0.6, 0.8, 1.0, and 1.2 m s-1
determination of leaf diameter (d)
determination of leaf heat capacity
calculation of gH using Eq. S8
calculation of gH using Eq. S2 and Eq. S7 with a and b being 1
substitution in Eq. S2 and rearranging to obtain Numeasured
rearranging Eq. S2 to obtain Nu (= Gr + 1.4.Re²)
log(Numeasured) vs. log(Gr + 1.4.Re²) (Fig. S2)
a and b (Tab. S1)
3. Experiment (actual experiment)
Measurement on a separate set of plants
Each individual leaf first measured in dark-adapted state at wind speeds of 0.0, 0.4, 0.8, and 1.2 m s-1 ,then measured in light-adapted state at wind speeds of 0.0, 0.4, 0.8, and 1.2 m s-1
Leaf geometry
gs
TL-Ta
TL-Ta images
τ
τ-images
Modelling hleaf
Evaluation and interpretation of τ with respect to leaf heat transfer processes

Fig. S4: Study workflow. Three experiments were performed during this study, of which experiment 1 and experiment 2 were dedicated to enable the modelling of the leaf water content (LWC), leaf heat capacity per unit area ($C\ A^{-1}_{leaf}$), and the leaf heat transfer coefficient (h_{leaf}), particularly the the convective heat transfer coefficient (h_H) and the leaf conductance to convective heat (g_H). Results of experiment 1 were required in experiment 2 in order to model g_H, which finally provided the parameters a and b required for Eq.8 (= Eq. S7). In an independently performed experiment (experiment 3), the impact of wind and illumination on the time constant (τ) was evaluated. Additionally, τ was compared to modelled h_{leaf}. For h_{leaf} modelling in the actual experiment, measured parameters, such as the mean leaf diameter (leaf geometry), stomatal conductance (g_s), and the difference between leaf temperature and ambient air temperature (T_L-T_a) were used in the model as well as the parameters derived from experiment 2. Finally, the measured τ (and τ-iamges) were evaluated and interpreted with respect to varying leaf heat transfer under varying ambient conditions (wind and light).

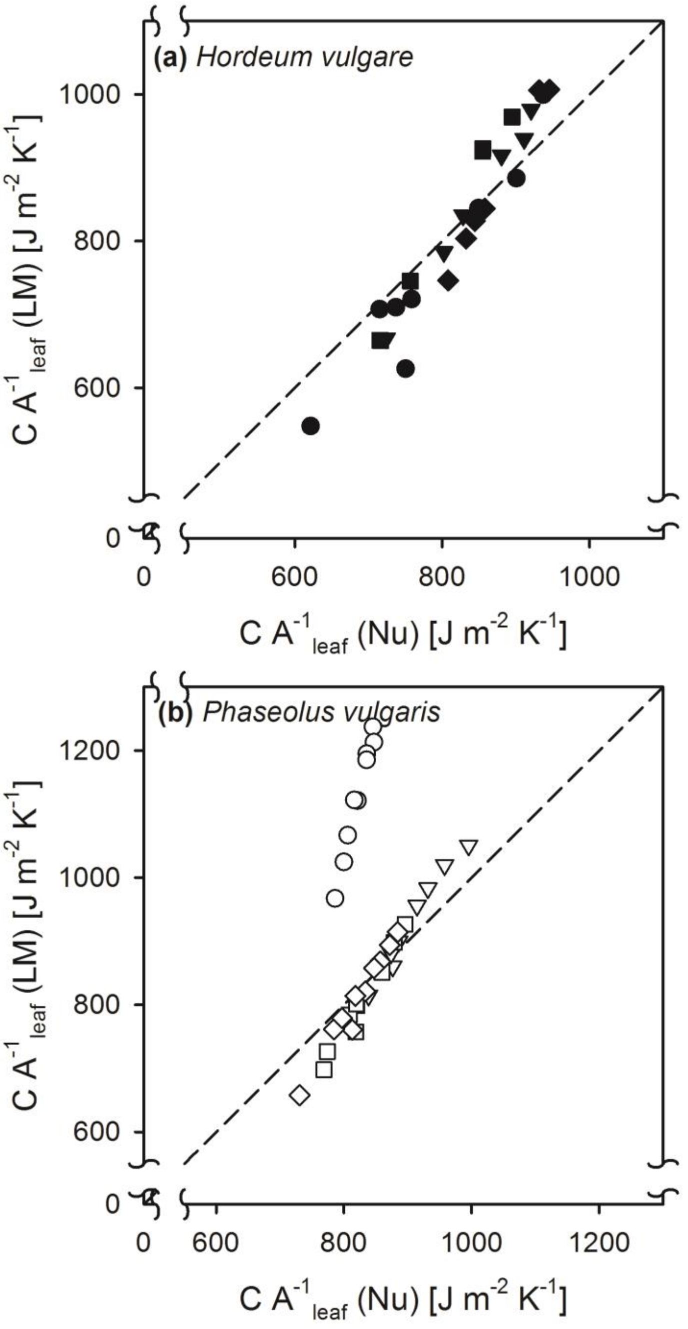

Figure S5: Comparison of two models for leaf heat capacity (C A^{-1}_{leaf}) prediction. C A^{-1}_{leaf} derived from linear regression of the relationship between time constant and leaf water content (LM) was compared to C A^{-1}_{leaf} derived from a dimensionless numbers model (Nu). **(a)** dark-adapted spring barley (*Hordeum vulgare*) leaves. **(b)** dark-adapted common bean (*Phaseolus vulgaris*) leaves. The modeled data are shown for measurements at four different wind speeds, 0.0 m s^{-1} shown by circles, 0.4 m s^{-1}

shown by triangles, 0.8 m s^{-1} shown by squares, and 1.2 m s^{-1} shown by diamonds. Dashed lines represent the one-to-one relationship.

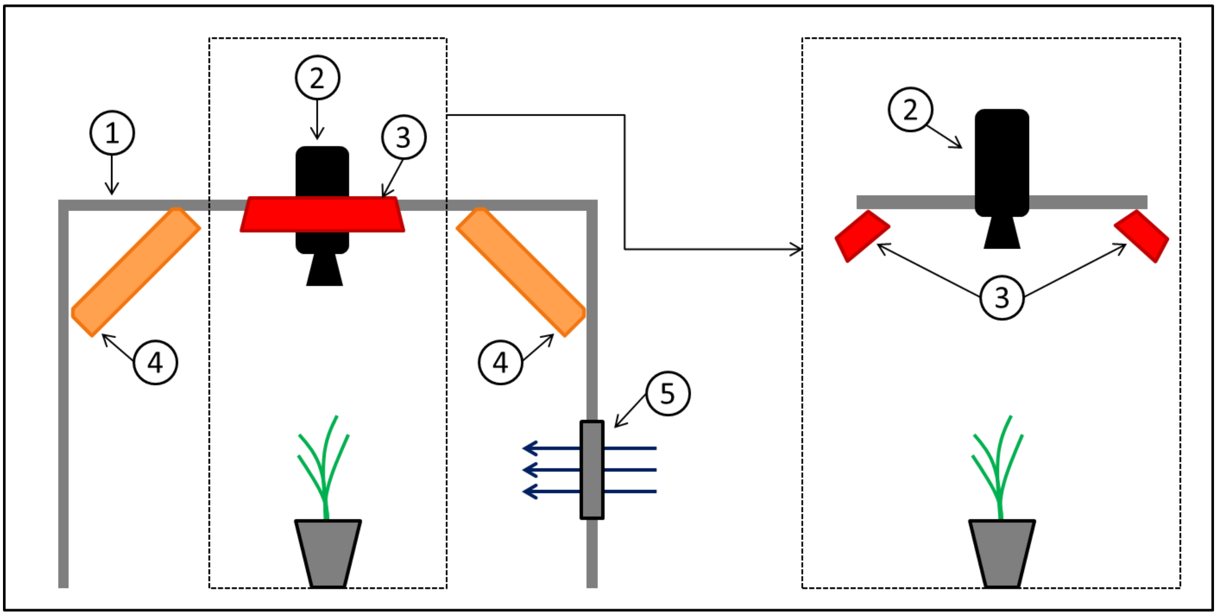

Figure S6: Experimental set-up. Thermal camera (VarioCAM ® hr head, Infratec, Germany) (2) and the near-infrared heater (Infralogic, Germany) (3) were installed in a 90° to the ground and the plants on metal profiles (1). LED panels (Photon System Instruments, Czech Republic) (4) provided light and a small ventilator (5) was installed at the height of the plants at a vertical bar.

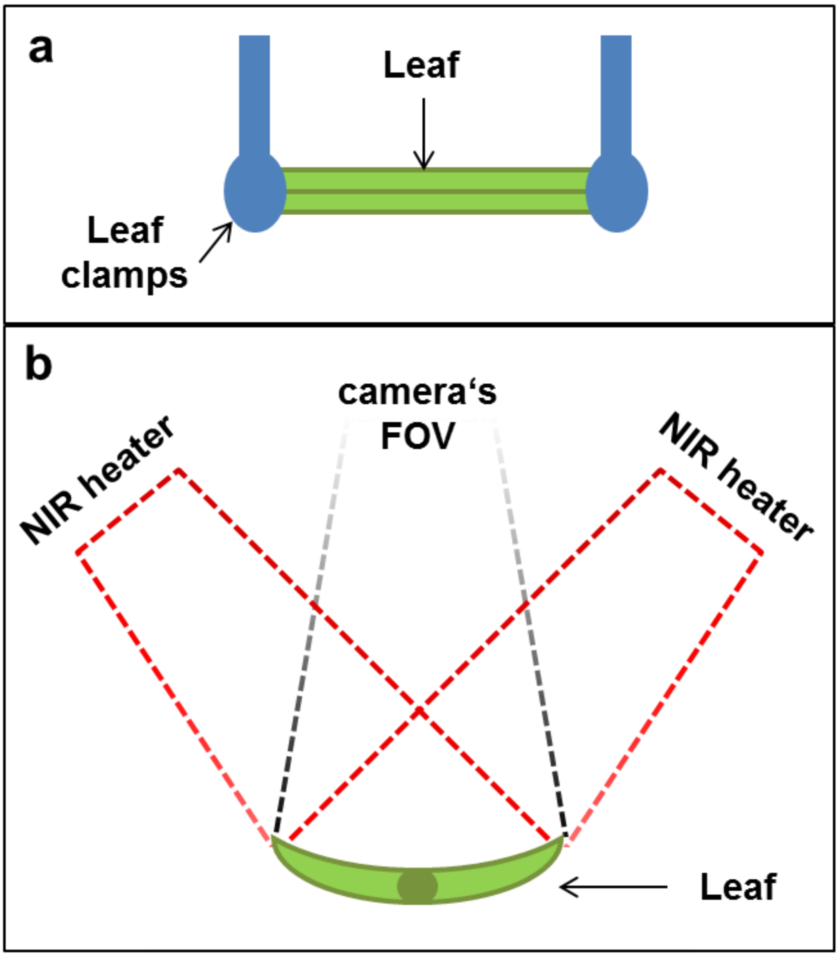

Figure S7: Experimental set-up. Leaves were fixed by leaf clamps, in order to provide a 90° angle to the camera's field of view (FOV).

Image heterogeneity and time constant gradients

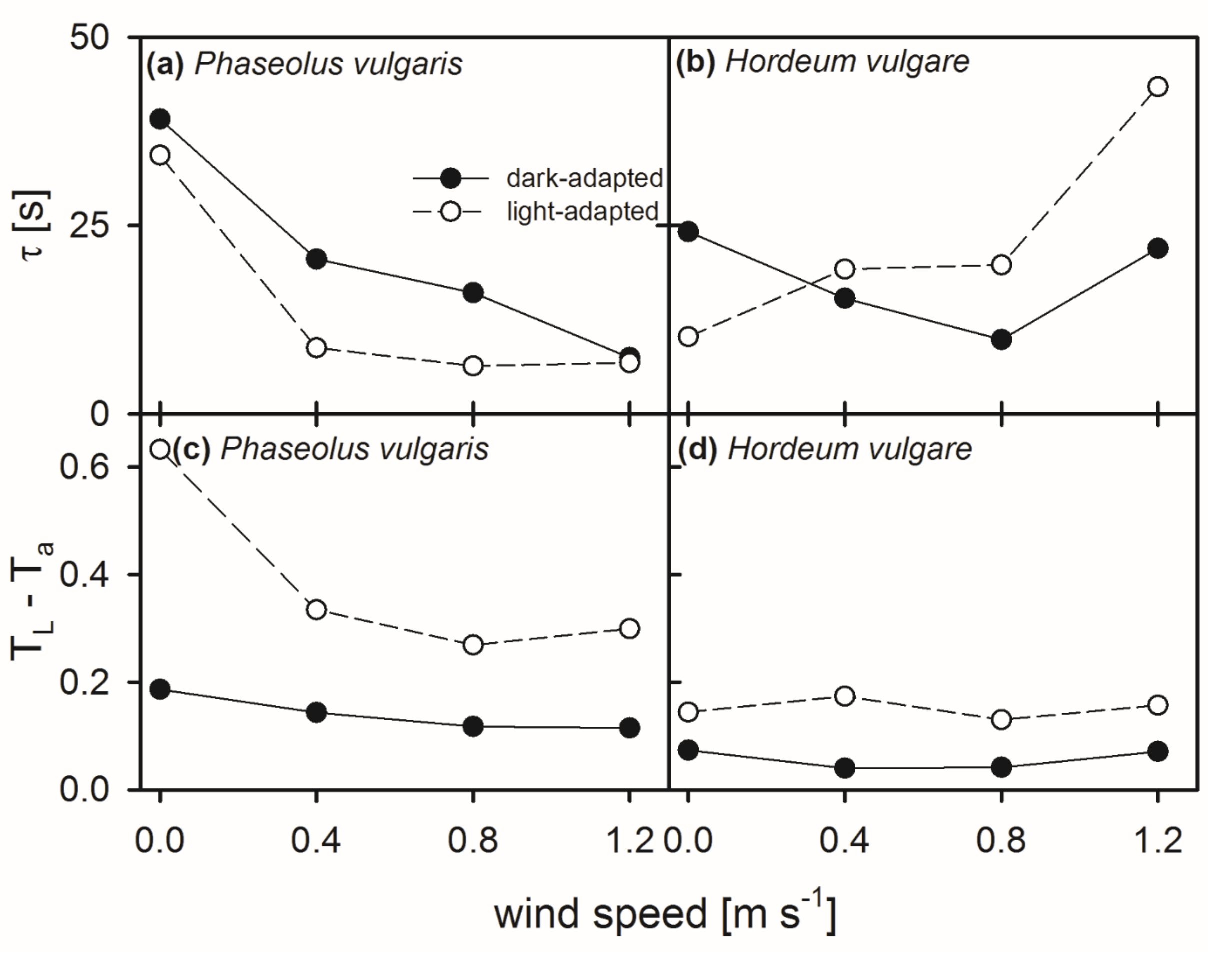

Figure S8: Standard deviation of time constant (τ) and leaf temperature to ambient air temperature (T_L-T_a) for a representative bean leaf in dependency of wind speed (Fig. 3a and 4a). Standard deviation was calculated for each image. Dark-adapted leaves are indicated by closed circles and light-adapted leaves are indicated by open circles.

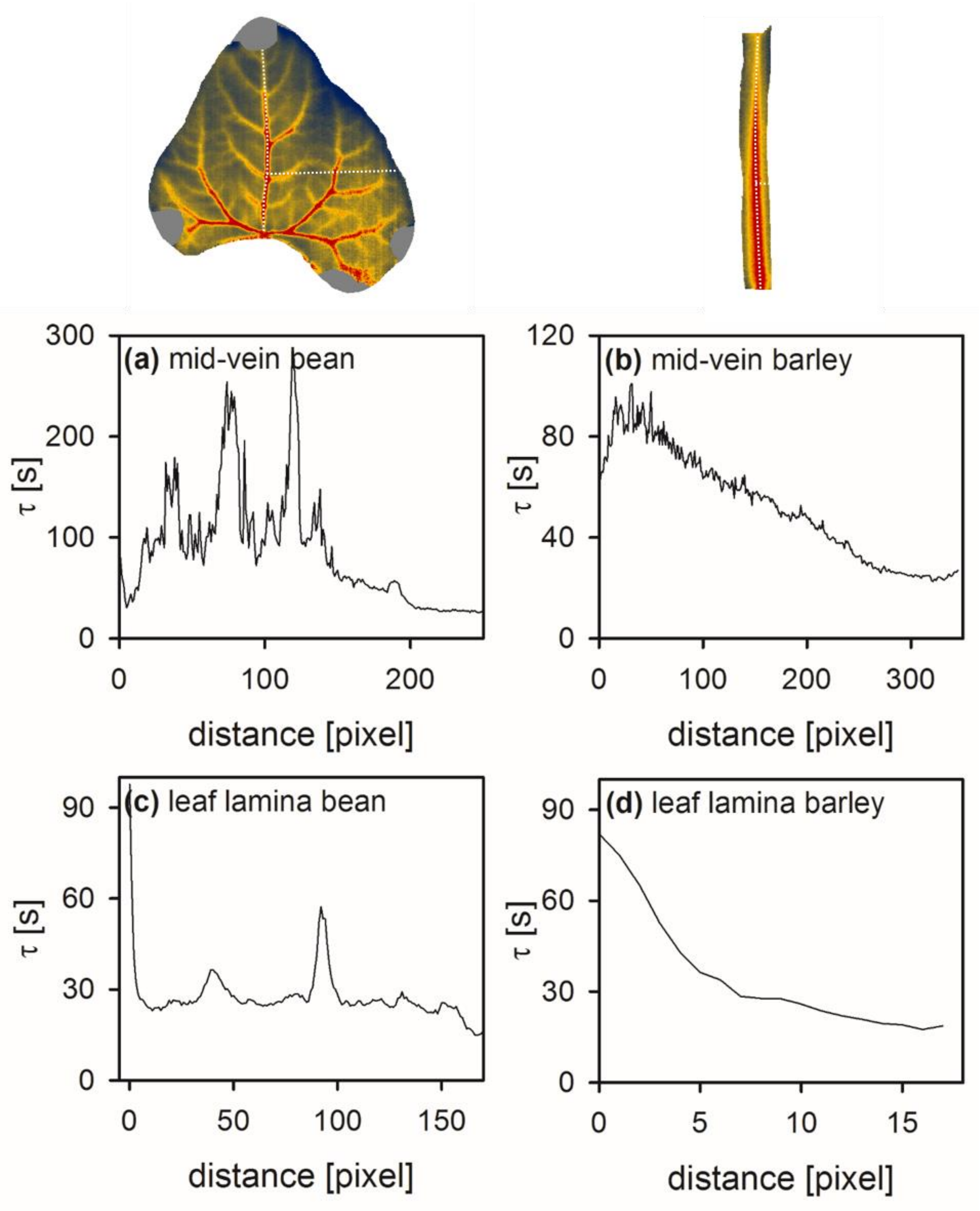

Figure S9: Gradient of time constant (τ) along the mid-vein and of leaf lamina form mid-vein to leaf edge. **(a)** and **(c)** show τ-gradients on a bean leaf, where the gradients were measured along the white dotted line as indicated in the images. **(b)** and **(d)** show τ-gradients on a barley leaf, where the gradients were measured along the white dotted line as indicated in the images.

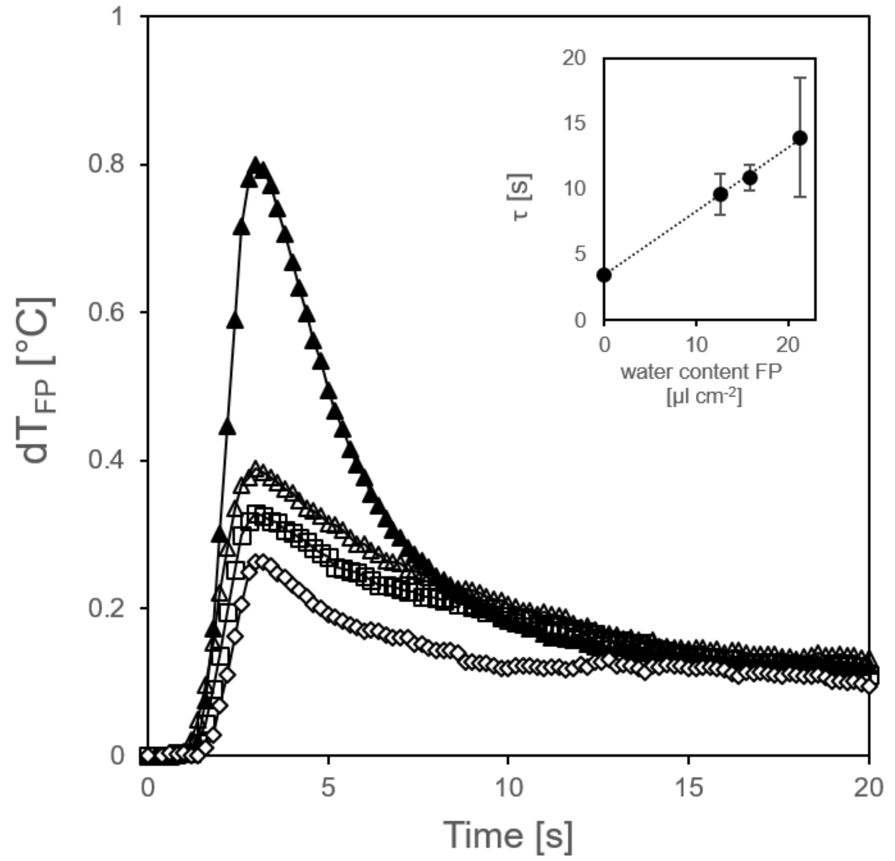

Figure S10: Heating/cooling curves of differently wetted filter papers. Temperature change before and after application of the heat pulse (dT_{FP}) was measured for 20 s. Filter papers were watered with either 0-µl cm^{-2} (solid triangle), 12.6 µl cm^{-2} (open triangle), 15.8 µl cm^{-2} (open square) or 21.3 µl cm^{-2} (open diamond). The graph at the top-right corner displays the observed linear relationship of calculated τ and the water content of the filter papers (mean ± SD, n=5). The temperature of the water used for wetting the filter papers was kept at 22°C using a water bath. Different volumes of water (0, 1200, 1500, 2000 µl] were added to round filter papers (area = 95 cm^2) by pipetting the water on the center of the paper. After homogenous distribution of the water, samples were transferred into the experimental setup as described in the manuscript. Measurements were conducted at wind speed of 0.0 m s^{-1}. Room temperature was around 22°C. Samples of different water content were measured randomized in order to minimize environmental effects.
